# The progress of gut microbiome research related to brain disorders

**DOI:** 10.1186/s12974-020-1705-z

**Published:** 2020-01-17

**Authors:** Sibo Zhu, Yanfeng Jiang, Kelin Xu, Mei Cui, Weimin Ye, Genming Zhao, Li Jin, Xingdong Chen

**Affiliations:** 1 State Key Laboratory of Genetic Engineering and Collaborative Innovation Center for Genetics and Development, School of Life Sciences, Fudan University, Shanghai, China; 2 Fudan University Taizhou Institute of Health Sciences, Taizhou, China; 3 Department of Epidemiology, School of Public Health, Fudan University, Shanghai, China; 4 School of Data Science, Fudan University, Shanghai, China; 5 Department of Neurology, Huashan Hospital, Fudan University, Shanghai, China; 6 Department of Medical Epidemiology and Biostatistics, Karolinska Institutet, Stockholm, Sweden; 7 Human Phenome Institute, Fudan University, 825 Zhangheng Road, Shanghai, 201203 China

**Keywords:** Gut microbiome, Metabolite, Neuropsychiatric disorders, Neurodegenerative disorders, Cerebrovascular diseases

## Abstract

There is increasing evidence showing that the dynamic changes in the gut microbiota can alter brain physiology and behavior. Cognition was originally thought to be regulated only by the central nervous system. However, it is now becoming clear that many non-nervous system factors, including the gut-resident bacteria of the gastrointestinal tract, regulate and influence cognitive dysfunction as well as the process of neurodegeneration and cerebrovascular diseases. Extrinsic and intrinsic factors including dietary habits can regulate the composition of the microbiota. Microbes release metabolites and microbiota-derived molecules to further trigger host-derived cytokines and inflammation in the central nervous system, which contribute greatly to the pathogenesis of host brain disorders such as pain, depression, anxiety, autism, Alzheimer’s diseases, Parkinson’s disease, and stroke. Change of blood–brain barrier permeability, brain vascular physiology, and brain structure are among the most critical causes of the development of downstream neurological dysfunction. In this review, we will discuss the following parts:

- Overview of technical approaches used in gut microbiome studies
- Microbiota and immunity
- Gut microbiota and metabolites
- Microbiota-induced blood–brain barrier dysfunction
- Neuropsychiatric diseases
  - ■ Stress and depression
  - ■ Pain and migraine
  - ■ Autism spectrum disorders
- Neurodegenerative diseases
  - ■ Parkinson’s disease
  - ■ Alzheimer’s disease
  - ■ Amyotrophic lateral sclerosis
  - ■ Multiple sclerosis
- Cerebrovascular disease
  - ■ Atherosclerosis
  - ■ Stroke
  - ■ Arteriovenous malformation
- Conclusions and perspectives

## Introduction

Since Rober Koch first developed a bacterial culture technique in the laboratory [1], Theodour Escherich identified the common gut bacillus *Escherichia coli* [2], and Élie Metchnikoff found an association between longevity and microbes from dairy food [3], an increasing number of commensal and pathogenic bacteria have been discovered and characterized as exerting a profound influence on human health and behaviors through food digestion, fermentation, metabolism, and vitamin production.

In the past five years, gut microbiota research has become a research“hot spot,” and more than 25,000 gut microbiota-related articles have been published (as of 1st Sep 2019). Using next-generation sequencing (NGS) approaches, large-scale studies such as the Human Microbiome Project (HMP) and the Metagenomics of the Human Intestinal Tract (MetaHIT) project have provided essential references regarding the microbiota composition in human bodies [4, 5]. Biomarkers in the gut are related to a variety of diseases, including metabolic disorders [6, 7], inflammatory bowel diseases (IBD) [8, 9], a variety of cancers [10], and even disorders of the central neural system.

**Fig. 1:**
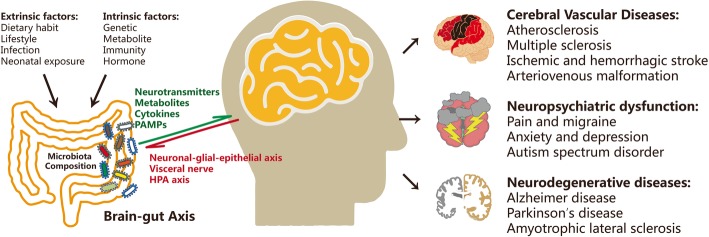
Dysregulation of the gut microbiota in brain disorders. Extrinsic and intrinsic factors shape the composition of gut microbiota and further contribute to brain disorders, including cognitive dysfunction, neurodegeneration, and cerebrovascular diseases

Researchers have identified alterations in the composition of the gut microbiota related to several symptoms or diseases, such as pain, cognitive dysfunction, autism [11, 12], neurodegenerative disorders, and cerebral vascular diseases [13]. The microbiota of different habitats contribute to bidirectional brain-gut signaling through humoral, neural, and immunological pathogenic pathways [14]. The central nervous system (CNS) alters the intestinal microenvironment by regulating gut motility and secretion as well as mucosal immunity via the neuronal-glial-epithelial axis and visceral nerves [15–19]. Extrinsic factors, including dietary habit, lifestyle, infection, and early microbiota exposure, as well as intrinsic factors such as genetic background, metabolite, immunity, and hormone, regulate the composition of gut microflora. On the other hand, bacteria react to these changes by producing neurotransmitters or neuromodulators in the intestine to impact the host CNS. These modulators include bacteria-derived choline, tryptophan, short-chain fatty acids (SCFAs), and intestinally released hormones such as ghrelin or leptin (Fig. 1).

Herein, we will review the progress in gut-brain axis studies and explain how changes in the gut microbiota alter cognitive function, cerebrovascular physiology, and the development of neurological and neuropsychiatric diseases.

## Technical approaches in gut microbiome studies

Current technologies do not allow the cultivation of all bacteria isolated from the gut. Two widely adopted culture-free approaches have been devised to effectively quantify and characterize the microbiome, i.e., targeted sequencing and metagenomic sequencing. Targeted sequencing is also referred to as marker gene sequencing [20] including 16S ribosomal RNA (rRNA), internal transcribed spacer (ITS), and 18S rRNA sequencing. In 1977, Woese et al. inferred phylogenetic relationships between prokaryotic organisms using the rRNA small subunit (SSU) genes; this approach was later proven to also work well in eukaryotes [21, 22]. Wilson and Blitchington showed a good agreement of the diversity and 16S rRNA sequences between quantitative cultured cells and direct PCR-amplified bacteria from a human fecal specimen [23].

Researchers now can use this powerful tool to sequence 16S rRNA for assessing microbial taxonomy and diversity from various human specimens.

However, 16S rRNA sequencing achieves only ~ 80% accuracy in the genus level and is not able to fully resolve taxonomic profiles at the species level or strain level, especially with short read lengths [24, 25]. Shotgun sequencing was then developed for the comprehensive profiling of the DNA from microbiota. In 1998, Handelsman et al. first coined the term “metagenome” when cloning DNA fragments from soil-derived biosamples into bacterial artificial chromosomal vectors [26]. In 2002, using the shotgun sequencing approach, Breitbart et al. sequenced a viral metagenome that did not carry rDNAs, and more than 65% of the species that they identified had not been reported previously [27]. The same year witnessed the isolation of the antibiotics Turbomycin A and B from a metagenomic library of soil microbial DNA by Gillespie et al. using a restriction enzyme approach [28]. In 2004, Tyson et al. sequenced biofilms using random shotgun sequencing and determined single-nucleotide polymorphisms at the strain level. It is now understood that the metagenome represents a collection of DNA from the environment, and shotgun sequencing has been widely adopted for metagenomic sequencing [29].

Other technologies including metatranscriptomics [30, 31], metaproteomics [32], and metabolomics [33, 34], can be applied to investigate RNAs, proteins, and metabolites in metagenome-wide association studies (MWAS). The MWAS approach has shown great potential not only in the identification of the microbiome taxonomy but also in the annotation of functions, pathways, and metabolism.

## Gut microbiome and host immunity

The human microbiota interacts with the host gut immune system for mutual adaptation and immune homeostasis by tolerating commensal antigens [35]. The perturbation of gut microbiota could lead to a higher incidence of autoimmunity and allergy [36]. Both innate immunity and adaptive immunity in the human gastrointestinal tract play critical roles as guardians that maintain pathogen-host homeostasis. In terms of innate immunity, pathogen-associated molecular patterns (PAMPs) were first suggested to describe the pathogenic targets recognized by pattern recognition receptors (PRRs) from the host innate immune system [37, 38]. PAMPs represent “molecular signatures” of a pathogen class, such as the highly conserved microbial structures consisting of lipopolysaccharides (LPS), lipoteichoic acid (LTA), CpG, or dsRNAs. Gut microbes and their derivatives constantly interact with PRRs in intestinal epithelial cells, immune cells in peripheral blood, and even cells in the CNS. Microbiota dysbiosis has been reported to trigger gut barrier dysfunctions, such as changes in tight junctions, mucous layers, and secretion of immunoglobulin A and intraepithelial lymphocytes [39, 40]. In the rodent model, hippocampal neurogenesis is proved to be controlled by the stimulation of toll-like receptors (TLRs). TLR2 is responsible for neurogenesis, while TLR4 exhibits a contrary function [41, 42]. By LPS binding, TLR4 inhibits retinal neurogenesis and differentiation via MyD88-dependent and NF-κB signaling pathways [43]. TLR2 activation also inhibits the proliferation of embryonic neural progenitor cells [44]. The downstream inflammatory cytokine TNF-α further reduces neurogenesis and induces the proliferation of astrocytes [45]. There is also evidence that TLR4 is related to the development of learning and memory [46, 47].

On the other hand, adaptive immunity routinely plays a crucial role in both anti-infection functions and the maintenance of microbiota-host homeostasis to prevent overreaction to harmless antigens. This balance is mainly accomplished by mutual regulation between regulatory T cells and TH17 intestinal lamina propria [48]. Regulatory T cells (Tregs) are crucial in the maintenance of immune homeostasis. The protective immunosuppression signals are delivered through GATA3, Foxp3, and IL-33 expression in regulatory T cells. SCFAs from dietary fiber are produced by Clostridia species, in particular, contributing to the activation and expansion of CD4+Foxp3+ Treg cells [49, 50]. The polysaccharide endocytosed by dendritic cells could promote expansion and differentiation of naïve T cells into Th1 and regulatory T cell subsets [51]. Gut microbiota-activated TH17 cells, characterized by IL-17A, IL-17F, and IL-22 secretion, are responsible for high-affinity IgA secretion, memory CD4+ T cell differentiation and anti-*Staphylococcus aureus* function [52–55]. However, dysbiosis of gut bacteria activates T and B cells and further influences the secretion and class switching of IgA in B cells, the differentiation of TH17 cells, and the further recruitment of dendritic cells, group 3 innate lymphoid cells (ILC3) and granulocytes [48]. When TH17 cells are induced by inflammatory signals such as IL-23 overexpression, these cells are likely to be associated with autoimmune diseases, including uveitis and encephalomyelitis [56–59]. Brain lesions in the hypothalamus-pituitary-adrenal gland axis (HPA) can alter intestinal immunity. In a mouse stroke model, a significant reduction of T and B cells in Peyer’s patches is observed 24 h after stroke [60]. In turn, impairment of the blood–brain barrier (BBB) following stroke is triggered by microbiota changes and immune dysregulation, further allowing brain infiltration of immune cells to react to CNS tissues [61].

## Microbiota altered blood–brain barrier and brain structure

BBB is a semipermeable barrier composed of specialized endothelial cells in the microvasculature [62]. The BBB separates the CNS from the peripheral blood [63]. There are a variety of disorders related to microbial-induced BBB dysfunction, which may cause anxiety, depression, autism spectrum disorders (ASDs), Parkinson’s disease, Alzheimer’s disease, and even schizophrenia [61, 64–66]. The exact mechanism whereby the microbiota affects BBB physiology remains unknown. Possible mechanisms include BBB modulation by gut-derived neurotransmitters and bacterial metabolites. Rodent models have shown that a loss of the normal intestinal microbiota results in increased permeability of the BBB, while a pathogen-free gut microbiota restores BBB functionality [67]. Metabolic diseases such as diabetes can result in increased permeability of the BBB and, potentially, further progression to Alzheimer’s disease with amyloid-β peptide deposition [64]. Microbiota dysbiosis has been found to alter the protective functions of the BBB, including regulation of permeability [67] through tight junction expression [68] or further behavioral changes [69].

The microbiota composition is also correlated with brain morphology. A germ-free mouse model demonstrated that the microbiota is required for the normal development of hippocampal and microglial morphology [70]. Structural magnetic resonance imaging (MRI) revealed that the patient’s cortical thickness is negatively correlated with the duration of Crohn’s disease [71]. The posterior regions and middle frontal gyrus have also been found to be reduced in adolescent patients [72]. The relative abundance of Actinobacteria is correlated with better organization of the amygdalar, hypothalamic, and thalamic microstructure according to MRI. The changes in structure are associated with better motor speed, attention, and cognitive test scores [73].

## Food and food-derived metabolites

As the second-largest metabolic organ in the human body, the gut harbors approximately 1.5 kg of colonized microbiota and metabolic mass from food [74]. The diet pattern plays an essential role in the composition of gut microflora and affects psychiatric conditions such as depression and anxiety [75, 76]. Diets rich in fruits, whole grains, vegetables, and fish have been proved to be beneficial to brain function such as the lower risk of dementia by reducing gut inflammation and neurodegeneration [77]. By far, several well-known “Mediterranean-like” dietary patterns have shown neuroprotective effects, i.e., Mediterranean diet (MeDi), Dietary Approach to Stop Hypertension (DASH) diet, and Mediterranean-DASH Intervention for Neurodegenerative Delay (MIND) diet [77, 78]. Patients with Alzheimer’s disease (AD) and atherosclerosis benefit from these “Mediterranean-like” diets [79]. On the other hand, diet rich in saturated fatty acids, animal proteins, and sugars have been shown to increase the risk of brain dysfunction. A high-fat diet (HFD), or namely “Western” diet, is normally regarded as detrimental to the brain [80]. Excessive intake of HFD is associated with increases in *Firmicutes* and *Proteobacteria* and a decrease in *Bacteroidetes* [81]. HFD also induces plasma and fecal levels of acetate, triggers the overproduction of insulin and ghrelin, and further promotes overfeeding [82]. Obesogenic effects and inflammation caused by HFD can be reduced by uptake of polyphenols from fruits, accompanied with an increase in *A. muciniphila.*

The food can quickly alter the microbiota composition in the gut. Changing to a high-fat or high-sugar dietary pattern from a low-fat or plant fiber-rich dietary pattern can shift of the microbiome even within one day [83]. A dog experiment revealed that the proportion of carbohydrate and protein was responsible for the change [84]. The *Bacteroides* abundance was reported to be associated with animal-derived protein and saturated fats, while *Prevotella* was associated with carbohydrates and simple sugars [85]. Vegetable-based diets can increase SCFAs, accompanied by elevated *Prevotella* and some fiber-degrading *Firmicutes* [86]. When fed with fructose, *Bacteroidetes* was significantly decreased in the mice model, while *Proteobacteria*, *Firmicutes,* and pathogenic *Helicobacteraceae* were significantly increased [87].

**Fig. 2:**
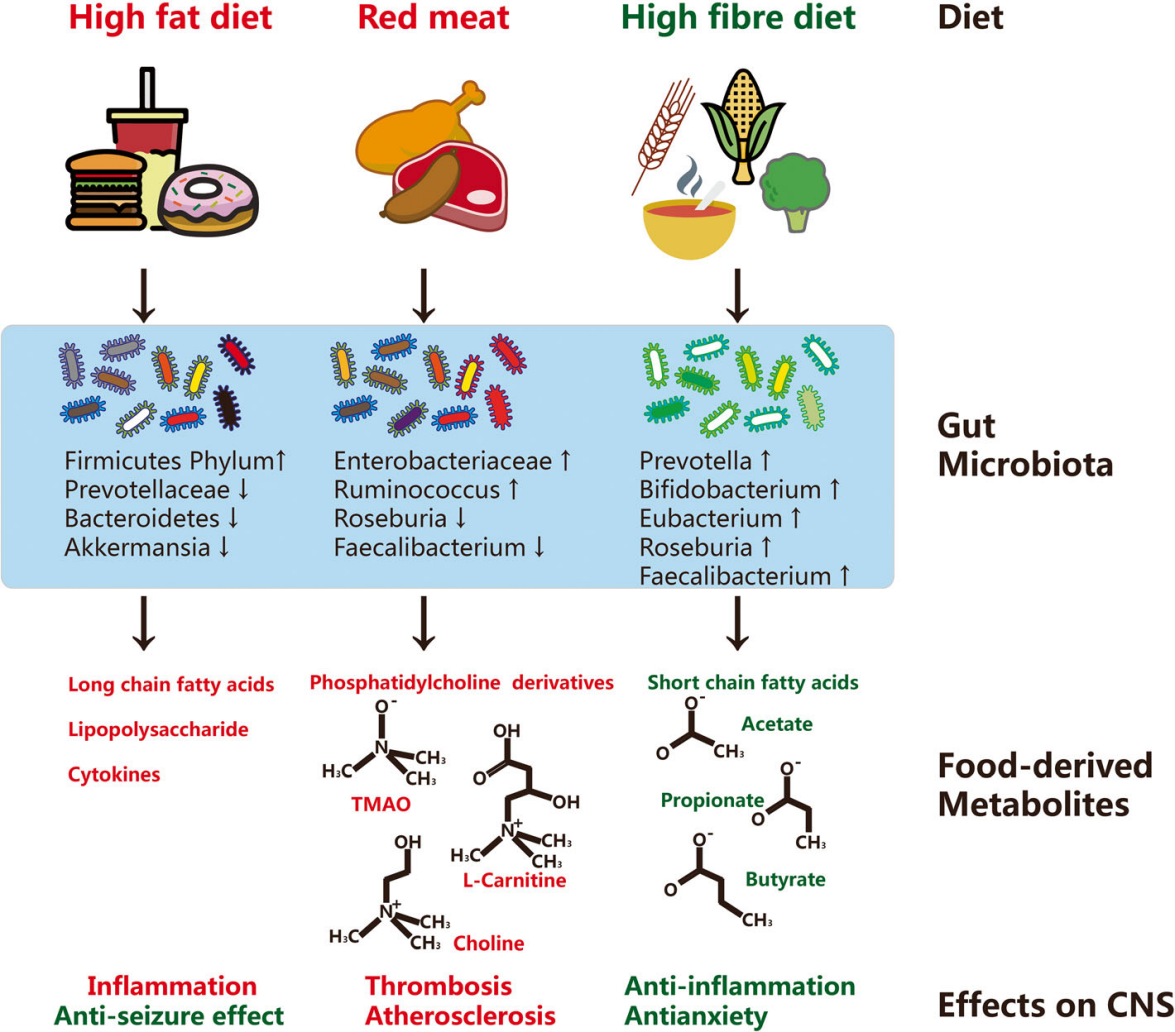
Dietary metabolism and roles of the gut microbiota. Dietary habit and food pattern result in the formation of gut microbiota and in turn modulate the host inflammation and thrombosis, by which the brain disorders are induced

Food patterns and dietary habits result in a change of brain physiology which can be explained by food-derived metabolites (Fig. 2). Metabolites derived from food play important roles in the pathogenesis of brain-related diseases. Recent findings showed the food-derived metabolites include not only SCFAs but also phosphatidylcholine, trimethylamine oxide (TMAO), L-carnitine, glutamate, bile acids, lipids, and vitamins. The food derivatives and microbe-fermented small molecular metabolites are released by gut microbiota into the blood which interacts with the host and further contributes to a variety of disorders, including brain diseases.

The metabolism of phosphatidylcholine which is rich in poultry, egg, and especially red meat, has been reported to involve a crucial biological interaction between the gut microbiota and host [88]. This pathway includes TMAO, a product of the oxidation of trimethylamine (TMA), which is produced during the metabolism of red meat-derived L-carnitine [89]. The levels of TMAO, betaine, and choline have been shown to serve as predictors in the diagnosis and prognosis of cardiovascular diseases [90–92]. These gut microbiota-derived metabolites induce and promote foam cell formation via cholesterol accumulation in macrophages. Recent studies have revealed that the *Clostridiales, Lachnospiraceae*, and *Ruminococcus* are highly correlated with the area of atherosclerotic lesion and TMAO levels in the plasma. Interestingly, an analog of choline, 3,3-dimethyl-1-butanol (DMB), was shown to inhibit the formation of TMAO in microbes [93]. An increased abundance of *Enterobacteriaceae* and *Ruminococcus* is found in atherosclerotic cardiovascular disease patients compared to controls, while butyrate-producing bacteria, including *Roseburia* and *Faecalibacterium*, are relatively depleted in these patients [94].

A highly ketogenic diet, with a high-fat, adequate-protein, and low-carbohydrate composition, has been shown to exert an anti-seizure effect [95]. This high-fat, low-carbohydrate pattern is associated with *Akkermansia* and *Parabacteroides* and results in a higher seizure threshold by reducing serum gamma-glutamylated amino acid levels and increasing brain GABA/glutamate levels [95]. On the other hand, a high-fat and high-cholesterol diet (HFHC) induces dyslipidemia and triggers the small intestine mucosal immune system, further promoting inflammation and altering the gut microflora [96]. The phylum *Firmicutes* is positively correlated with carbohydrate oxidation, while *Bacteroidetes* exhibits a negative correlation. Herbal dietary supplements such as Rhizoma Coptidis alkaloids have been shown to alleviate hyperlipidemia in a mouse model by modulating the gut microflora and bile acid pathways [97].

A high-fiber diet has been reported to improve brain health through multiple effectors [98]. In the mammalian gut, dietary fiber is degraded by bacteria to produce long-chain fatty acids (LCFAs) and SCFAs as metabolites [99]. LCFAs, including lauric acid, promote the differentiation of TH17 cells. The LCFAs are known as trigger factors in the establishment of autoimmune encephalomyelitis model [100]. In contrast, SCFAs usually not only serve as energy sources for epithelial cells but also recruit and expand regulatory T cells and release modulatory cytokines [101]. SCFAs are mainly composed of acetate (40–60%), propionate (20–25%) and butyrate (15–20%) [102, 103]. Acetate and propionate are produced by the Bacteroidetes phylum in particular, while species of the Firmicutes phylum preferentially produce large amounts of butyrate [104]. Systemic loss of SCFAs, especially acetate, is likely to exacerbate inflammatory reactions in germ-free mice, whereas direct acetate drinking is helpful to ameliorate disease by decreasing the level of the inflammatory mediator myeloperoxidase [105]. Propionic acid protects the mouse model from autoimmune colitis better than other SCFAs through the induction of Treg cells [50, 100]. A high-fiber diet inducing butyrate production can protect the brain well and enhance neuron plasticity in a neurogenesis model [98]. It is worth noting that SCFA can manipulate the acetylation and methylation state and further regulating the host’s gene expression and metabolic processes [106]. For example, lower abundances of SCFAs are shown associated with a lower degree of chromatin acetylation [107].

Dietary tryptophan is an essential source of an aryl hydrocarbon receptor (AHR) agonist that limits CNS inflammation by reducing both astrocyte and microglial pathogenic activities and experimental autoimmune encephalomyelitis (EAE) development [108, 109]. Tryptophan is usually metabolized by a bacterial tryptophanase (TnAse) from the gut microbiota to generate indole, indole-3-propionic acid (IPA), and indole-3-aldehyde (IAld). Indole is a precursor of the AhR agonist indoxyl-3-sulfate (I3S) [110]. Recently, dietary tryptophan was reported to protect mice against multiple sclerosis [108]. However, it has also been indicated that a high level of indole in a rat model increases the likelihood of developing brain dysfunctions, including anxiety and mood disorders [111].

Szczesniak et al. found that bacteria such as Faecalibacterium, Alistipes and Ruminococcus were correlated with depression, as well as the level of isovaleric acid, a type of volatile fatty acid (VFA) [112]. These associations probably occur because gut-derived VFAs can pass the BBB and further interact with synaptic neurotransmitters. Interestingly, Wu et al. revealed that although the plasma metabolome of vegans is significantly different from that of omnivores, the microbiota composition of these groups is similar [113].

One of the dietary tyrosine metabolites, 4-ethylphenylsulfate (4-EPS), is able to induce autism spectrum disorder (ASD)-like behaviors. However, following injection with *Bacteroides fragilis*, the gut microbiota produces reduced levels of neurotoxic metabolites, including 4-EPS, serum glycolate, and imidazole propionate, correcting gut permeability and ameliorating anxiety-like behavior [114].

## Neuropsychiatric dysfunction

Mood, memory, and cognition were originally thought to be exclusively regulated by the CNS due to a variety of extrinsic factors such as hormonal fluctuations [115]. It is now becoming clear that many non-nervous system factors, including the immune system and the resident bacteria of the gastrointestinal tract, regulate not only our feelings and how we form, process, and store memories but also cognitive function-related microstructure and morphology. Psychogastroenterological studies have been performed to analyze the microbiota regulating resilience, optimism, mindfulness, and self-regulation and mastery [116]. Alteration of the gut microbiota regulates not only the synthesis of metabolites but also different neuroactive molecules and central neurotransmitters, such as melatonin, gamma-aminobutyric acid (GABA), serotonin, histamine, and acetylcholine [117]. Germ-free and antibiotic rodent models have shown that microbiota exposure can induce depression, anxiety and stress, decreases in social communication, increases in exploratory activity, and memory deterioration (Table 1).

**Table 1:** Rodent models in mood disorders and cognitive function research

Animal	Model type	Phenotype and effect (vs. controls)
NMRI mice	Germ-free	Increased motor activity and reduced anxiety [61]
Wistar rat	Application of probiotics: *Lactobacillus helveticus* R0052 and *Bifidobacterium longum* R0175	Decreased stress-induced gastrointestinal discomfort and anxiolytic effect [118]
Balb/c mice	Application of probiotics: *Lactobacillus rhamnosus* (JB-1)	Increase of corticosterone, GABA receptor, *N*-acetyl aspartate and glutamate; reduced stress-induced corticosterone and anxiety- and depression-related behavior [119, 120]
Balb/c mice	Germ-free, SPF and *B. infantis* mice	GF mice exhibited exaggerated HPA stress response and decreased BDNF compared to SPF mice, but can be reversed with *B. infantis* [121]
Swiss Webster mice	Germ-free	GF mice have a anxiolytic behavior, increased BDNF and reduced serotonin 1A receptor in dentate gyrus of the hippocampus [122]
Balb/c mice and AKH mice	*T. muris* infection and application of probiotics *B. longum* NCC3001	Infection with *T. muris* induced anxiety-like behavior and decreased level of BNDF. Treatment with *B. longum* reverses the effect and normalizes BDNF level [123]
Balb/c mice and NIH mice	Germ-free and SPF with antibiotics	Increased exploratory behavior and hippocampal expression of BDNF in hippocampus [124]
Stress-sensitive F344 Rat	Germ-free and SPF	Decreased social interaction in GF mice [125]
Swiss Webster mice	Germ-free	Significant social impairments and decreased social preference in GF mice [126]
Wistar rat	Application of antibiotic	Reduced social interactions in offsprings when antibiotic is applied peri-conceptionally [127]
Swiss Webster mice	Germ-free	Increased exploratory in GF mice [122]
Swiss Webster mice	Application of antibiotic	Increased exploratory behavior in antibiotic-treated mice [126]
C57BL/6 mice	Application of antibiotic	Decreased working memory in antibiotic-treated SPF mice; no effect on spatial memory [128]
Swiss Webster mice	Germ-free	Decreased working memory in GF mice [129]
C57BL/6 mice	SPF	Decreased working memory in antibiotic-treated SPF mice [130]
C57BL/6 mice and CF1 mice	*Citrobacter rodentidum* infection	Increase anxiety and memory dysfunction [129, 131]

Early life is an important period in the development of the nervous system of the host. The microbiota colonizes the host immediately after birth within a few weeks and forms organ-specific niches [132, 133]. The initial commensal microbiota gradually forms similar communities in adults in the following 2–3 years [134]. However, the process can be influenced by inflammation during this stage. As an important modulator of neurogenesis, microglia may play an inflammatory and detrimental role in neural development when activated by LPS. Such effects result in abnormal host behaviors and learning impairments later in adulthood [135]. The microbiota can facilitate the development of host neurological functions (i.e., the development of cognitive functions and memory). In a mouse model, long-term exposure to a western diet has been shown to not only cause obesity but also trigger systemic inflammatory responses to LPS and to further induce cognitive deficits such as poorer spatial memory [136]. Magnusson et al*.* indicated that high-energy food alters the composition of the microbiota and impacts working memory and cognitive flexibility, resulting in poorer long-term memory formation [137]. Infection by bacteria such as *C. rodentium* is another trigger that may generate stress-induced memory dysfunction in a rodent model [129].

### Stress and depression

The gut microbiota has been shown to play critical roles in the pathogenesis of depression and anxiety-like behavior [138, 139]. Abnormal HPA hyperactivity in response to stress is associated with depressive episodes. Sudo et al. found that the HPA stress response is hyperactive in germ-free mice, but this hyperactivity can be reversed by *Bifidobacterium infantis* [121]. Plasma ACTH and corticosterone levels have been observed to be higher in GF mice than SPF mice when responding to stress. However, Diaz et al. revealed that GF mice exhibit increased plasma levels of tryptophan and serotonin compared with SPF mice [61], which is correlated with increased motor activity and reduced anxiety. Neufeld et al.’s results also demonstrated that GF mice exhibit anxiolytic behavior via increased expression of brain-derived neurotrophic factor (BDNF) and reduced serotonin 1A receptor levels in the hippocampus [122]. Anxiety and depression are typical complications in irritable bowel syndrome (IBS) patients, who show a higher prevalence of these conditions than healthy controls [140]. In addition to anxiety and depression, IBD patients exhibit mild verbal memory dysfunctions. The VFA isovaleric acid has been regarded as an important mediator of depression [112]. Gut-derived isovaleric acid can cross the BBB and interfere with synaptic neurotransmitter release. Isovaleric acid is positively correlated with saliva cortisol, which is strongly associated with depression in boys [141]. Bravo et al. have shown that GABA receptor expression in different brain areas can be altered by chronic treatment with *Lactobacillus rhamnosus.* This treatment further reduces stress-induced corticosterone levels and depression-like behaviors [119]. Stress-induced reduction of hippocampal neurogenesis can also be prevented by a probiotic combination of *Lactobacillus helveticus* strain R0052 and *Bifidobacterium longum* strain R0175 [142]. De Theije et al. showed that the effect of probiotics is highly strain specific. The administration of *Campylobacter jejuni* promotes depressive- or stress-like behaviors, while *Bifidobacterium* strains exert a decreasing effect on these behaviors [143].

### Autism spectrum disorder

ASDs are characterized by abnormal communication and social behaviors, which begin in early childhood neurodevelopment. Gut problems or a history of gastrointestinal perturbations such as infection and anti- or pro-biotic intake in early childhood contributes to the disease development [144–146]. Gastrointestinal discomfort in ASD patients is usually thought to have a neurologic rather than a gastroenteric cause [147, 148]. However, recent studies have revealed that gut microflora alteration-related gastrointestinal symptoms are associated with mucosal inflammation [148, 149]. Anti-inflammatory and SCFA-synthesizing species such as *Faecalibacterium* species are decreased in ASD patients compared to controls [150]. Oral administration of *Bacteroides fragilis* (a commensal bacterium) can correct intestinal epithelial permeability caused by dysbiosis, leading to the modulation of several metabolites and ameliorated symptoms of ASD [114]. The Firmicutes/Bacteroides ratio and the composition of Fusobacteria or Verrucomicrobia are associated with ASDs. Lower levels of *Bifidobacteria* species, mucolytic bacteria, and *Akkermansia muciniphila* are found in children with ASD, while *Lactobacillus*, *Bacteroides*, *Prevotella*, and *Alistipes* are present at higher levels [145, 151, 152]. However, *Desulfovibrio* species, *Bacteroides vulgatus*, and Clostridia are over-represented in children with ASD with gastrointestinal symptoms compared with normal subjects with similar GI complaints [153, 154]. One hypothesis regarding the reoccurrence of ASD symptoms after oral vancomycin treatments is that Clostridia undergo spore formation to avoid eradication [145]. Although there is little direct causal evidence that the microbiota can cure ASD, the use of probiotic strains of *Lactobacillus* species and *Bifidobacterium* species has been shown to ameliorate gastrointestinal symptoms in children with ASD [119, 155]. Hsiao et al.’s research suggested that oral treatment with *Bacteroides fragilis* in a mouse model altered the microbial composition, improved gut permeability, and reduced defects in communicative and anxiety-like behaviors [114].

### Pain and migraine

Pain is common in the general population, and the microbiota has shown to impact several types of pain, including spinal visceral pain in IBS [138, 156] and small intestinal bacterial overgrowth (SIBO) [157] as well as migraine [158] and sometimes headache [159]. Administration of antibiotics may induce gut dysbiosis and alter host-bacterial interactions, leading to colonic sensory and motor changes in a mouse model. These effects can be reflected by nociceptive markers such as CB2 and TLR7 [160]. Amaral et al. showed that inflammatory hypernociception induced by LPS, TNF-α, IL-1beta, and the chemokine CXCL1 is reduced in germ-free mice. This effect was similar to the induction by prostaglandins and dopamine [161]. It has been revealed by meta-analyses that *Helicobacter pylori* infection is associated with migraine [162]. Faraji et al. performed a randomized blinded clinical trial that demonstrated improvement of migraine associated with *H. pylori* eradication [158]. A recent study revealed that gut microbiota dysbiosis-induced chronic migraine-like pain are accomplished by up-regulating TNF-α level [163].

On the other hand, visceral pain can be effectively alleviated by probiotic treatment in animal models. Rousseaux et al. found that analgesic functions could be achieved by taking specific *Lactobacillus* strains orally. The resulting morphine-like effect was shown to be mediated by enhanced expression of intestinal epithelial μ-opioid and cannabinoid receptors [164]. Probiotic *B. infantis* 35624 and *Lactobacillus farciminis* were shown to exert visceral antinociceptive effects by altering central sensitization in rat models [165, 166]. *L. farciminis* can also attenuate stress-induced Fos expression in the spine, which explains the antinociceptive effect of this probiotic. In a clinical-alimentary study, O’Mahony et al. treated patients with probiotics and found that *Lactobacillus salivarius* UCC4331 significantly reduced pain and discomfort during the administration phase for one week, while *B. infantis* 35624 reduced pain and discomfort scores during both the administration and washout phases [167].

## Neurodegenerative diseases

Neurodegenerative diseases are a collection of neurological diseases that are characterized by progressive loss of neurons, including AD, Parkinson’s disease (PD), amyotrophic lateral sclerosis (ALS) and multiple sclerosis (MS) [168]. Each of the neurodegenerative diseases has been reported to have unique pathologies and clinical features. Nevertheless, neuroinflammation and higher intestinal permeability are common characteristics of them [169]. Some of the peripheral inflammation factors such as TNF-α, iNOS, and IL-6 have been validated in the pathogenesis of the neurodegeneration in CNS [170, 171]. In this chapter, we will discuss how functional gastrointestinal disorders are critically linked to these neuropathies.

### Parkinson’s disease

PD is a typical neurodegenerative disorder affecting more than 1% of the population over 65 years of age [172]. PD is thought to be caused by the interaction between environmental and genetic risk factors. This neuropathology is characterized by motor deficits and non-motor symptoms (NMS), which ultimately have an impact on quality of life [173].

Recently, the gut microflora has drawn increasing attention with respect to how it may be implicated in PD [173]. A variety of enteral dysfunctions are associated with PD, such as SIBO, malnutrition, *H. pylori* infection, and constipation [174]. In terms of the role of GI tracts pathology in PD, a higher frequency of α-synuclein detection is found in the patients than in controls from many researches [175]. Animal studies validated that resection of vagus nerve can stop transmission of α-synuclein from gastro intestine to the CNS [66, 176]. Bowel inflammation can also trigger neuroinflammation to promote dopaminergic neuronal loss in the rodent substantia-nigra tissue [177]. Forsyth and colleagues found that “leaky guts” and bowel inflammation affect the progression of PD [178]. The increased colonic permeability was found to be correlated with increased α-synuclein and *E. coli* accumulation in the sigmoid of PD patients [179].

Counts of butyrate-producing and anti-inflammatory bacterial genera such as *Blautia*, *Coprococcus*, and *Roseburia* are significantly lower in PD patients, while those of LPS-producing genera *Oscillospira* and *Bacteroides* are significantly higher [180]. The pro-inflammatory genus *Ralstonia* is significantly abundant in PD patient mucosae, implying that the inflammatory gut barrier is pathogenic. Scheperjans et al. revealed that the altered intestinal microbiota and the related motor phenotype could be applied to the diagnosis of PD [181]. The relative abundance of Prevotellaceae is significantly reduced in PD and has been validated as a very sensitive biomarker for PD diagnosis. A model based on multiple bacterial families and constipation status was shown to present 90.3% specificity in PD diagnosis. Ingestion of fermented milk for 4 weeks was proven to be effective in improving PD complications such as constipation [182]. Two studies have shown that anti-TNF therapy and immunosuppressants reduced PD risk [183, 184]. However, there is very limited clinical evidence of the beneficial effects of probiotics in the treatment of PD and further evidences are needed.

### Alzheimer’s disease

AD, which is the most common degenerative disease, is characterized by a decline in cognitive skills, including memory, language, and problem solving, eventually resulting in dementia [185]. AD is pathologically characterized by neuroinflammation, accumulation of beta-amyloid (Aβ) plaque, and neurofibrillary tau tangles in the brain. Aβ (Aβ40 or Aβ42) are cleaved products from the amyloid precursor protein (APP). The Aβ polymerizes into fibrils in the CNS by self-aggregation and further triggers inflammation and neurotoxicity. Recent studies have demonstrated the role and impact of microbial dysfunction and infection on the aetiology of AD, especially the activation of neuroinflammation and the formation of amyloids.

Neuroinflammation factors, including IL-1beta, IL-6, and TNF-alpha, have been observed in AD patients [186, 187]. Gareau et al.’s work implied that memory loss and dysfunction are exacerbated by infection under exposure to acute stress [129]. Released LPS triggers the inflammation and promotes amyloid fibrillogenesis in the brain [188]. The microglia can recognize amyloid molecules by TLR4 and TLR2 for clearance. Interestingly, signaling of myeloid differentiation primary response 88 (MyD88) from microglial TLR2 is responsible for activation of TNF-α and nuclear factor kappa B (NF-κB) which also induces the formation of Aβ by promoting α-secretase and β-secretase, respectively [189].

As a generic term, amyloid denotes any insoluble, aggregation-prone, and lipoprotein-rich deposit that resembles carbohydrate starches [190]. Bacterial metabolites and products have recently been shown to worsen AD. In AD patients, bacteria-derived amyloids (curli, tau, Aβ, α-syn, and prion) can function as initiators to cross-seed and aggregate host amyloids [191]. Amyloids such as CsgA and Aβ42 can exhibit cerebral deposition and trigger a cascade of AD-related pathological events despite their dissimilarity of sequences [192]. It is reported that chronic *H. pylori* could trigger the release of both inflammatory mediators and amyloids in AD patients [193, 194]. *H. pylori* filtrate was shown to have the ability to induce the hyperphosphorylation of tau protein in a cell model [195]. *E. coli* has been reported to produce extracellular amyloids known as curli fibres, a major subunit of CsgA. Other amyloids produced by microbes include CsgA produced by *Salmonella* spp., FapC by *Pseudomonas fluorescens*, MccE492 by *Klebsiella pneumonia*, phenol-soluble Modulins by *Staphylococcus aureus*, TasA by *Bacillus subtills*, and Chaplins by *Streptomyces coelicolor* [190, 196, 197]. Harach et al. found a remarkable shift in the gut microbiota in fecal samples from an APP-transgenic mouse and reported that the microbiota contributed to the development of this neurodegenerative diseases. Intestinal germ-free APP transgenic mice were found to have a reduction of cerebral amyloid pathology compared to controls [198]. More recently, *Chlamydia pneumoniae* infection in astrocytes has been demonstrated to be involved in the generation of β-amyloid, which promotes AD [199]. Ingestion of probiotics has been reported to be beneficial in AD. Azm S et al. demonstrated that *Lactobacilli* and *Bifidobacteria* were effective in ameliorating memory and learning dysfunction in a β-amyloid-injected rodent model [200]. Wang and Liang et al. revealed that *Lactobacillus fermentum* NS9 and *Lactobacillus helveticus* NS8 alleviated ampicillin-induced spatial memory impairment and improved the spatial memory of chronic restraint stress [201, 202]. In a randomized clinical trial of probiotic treatment conducted in 60 AD patients, Akbari et al. showed that after 12 weeks of administration of *Lactobacillus* and *Bifidobacterium* species via fermented milk, mini-mental state examination (MMSE) scores improved significantly. Improvements in glucose and lipid metabolism were also observed, which were thought to enhance the cognitive assessment scoring [203]. The elimination of *Helicobacter pylori* by triple eradication therapy results in improvement of cognition parameters in AD patients [204]. It is promising that the use of pro- or antibiotics could be future therapeutic agents for AD. It has been demonstrated in a recent study that exercise and probiotics could reduce Aβ plaques in the hippocampus, improve cognitive performance, and finally, attenuate the development of Alzheimer's disease in the mouse model [205].

### Amyotrophic lateral sclerosis

ALS is a fatal neurodegenerative disorder that affects the brain and spinal cord neurons and typically results in death. Most ALS patients die within 3 to 5 years due to respiratory paralysis [206]. The pathogenesis of ALS is proposed to be outcomes of genetic-environmental interactions. Studies on interactions between innate immune response and LPS have provided essential evidences of ALS pathogenesis [207–209]. More than a decade ago, it was proposed that gut-derived neurotoxins, including tetanus and botulinum toxins produced by *Clostridia* species, cause ALS [210, 211]. “Leaky gut” is also likely to be responsible for ALS [212]. More recently, Wu’s ALS mouse model revealed that the tight junction structure was damaged and that gut permeability was increased. Gut dysbiosis is also found in ALS mice, particularly in terms of reduced levels of butyrate-producing bacteria, including *Butyrivibrio fibrisolvens* and *E. coli* [213]. In the gut of ALS patients, butyrate-producing *Oscillibacter*, *Anaerostipes*, and *Lachnospira* counts were found to be reduced, while that of glucose-metabolizing *Dorea* was significantly increased [214]. Brenner et al. found the richness of OTUs to be significantly higher in an ALS group compared with a control group. However, the relative ratio of *Bacteroidetes/Firmicutes* did show a significant difference between ALS patients and controls [215]. Supplementation of the diet with 2% butyrate in drinking water in an ALS mouse model resulted in improved gut integrity and survival [216]. Mazzini et al. revealed unique bacterial profiles in ALS patients compared to the controls. Higher *E. coli* and *Enterobacteria* abundance, and lower *Clostridium* was found in ALS participants [217]. These results imply that anti-inflammatory SCFAs produced by gut microbiota are potential therapeutic agents affecting ALS progression. Mazzini et al. further conducted a clinical trial of bacteriotherapy aimed at understanding the effect of *Lactobacillus* strains [217]. However, more direct evidence and results are needed to clarify how the gut microbiota improves or aggravates ALS.

### Multiple sclerosis

MS is a type of autoimmunity-induced neurodegenerative disease in the spinal column and CNS. The environmental factors contribute profoundly to pathogenesis of this demyelinating disease, including obesity, smoking, viruses, and vitamin D [218]**.** Alterations in the microbiome and prevalence of “leaky gut” have been found in MS patients and experimental autoimmune encephalomyelitis (EAE) animal, the most commonly used inflammatory demyelinating disease model [219, 220]. The immunological changes in EAE are characterized by increasing proinflammatory cell infiltration and impaired Treg function [221]. The favorable gut microbiota can regulate permeability of BBB, limit astrocyte pathogenicity, and activate microglia [222]. In the EAE model, the *Bifidobacterium* and lactic acid-producing bacteria such as of *Lactobacillus* are able to reduce severity of EAE symptoms [223, 224]. The abundance of Archaea is high in MS while *Firmicutes* and *Bacteroidetes* phyla are lower or even depleted [218]. The *Bacteroides* and *Clostridia* species are especially responsible for the induction of FoxP3+ Tregs in suppression of inflammation [222]. One small longitudinal study found that the gut microbiota profiles, especially *Fusobacteria* were associated with future relapse risk in MS [225]. *Psuedomonas*, *Haemophilus*, *Blautia*, and *Dorea* genera were detected to be increased in MS patients, while *Parabacteroides*, *Adlercreutzia* and *Prevotella* genera were much lower [219]. *Clostridiales* order were restored after MS treatment with glatiramer acetate including *Bacteroidaceae*, *Faecalibacterium*, *Ruminococcus*, *Lactobacillaceae*, and *Clostridium* [226]. Ochoa-Reparaz et al. unraveled that oral administration of antibiotics can delay the pathogenesis of EAE. However, intraperitoneal injection did not significantly impact the outcomes [227]. This implies changes in intestinal microbiota ecosystem are associated with MS development. Other studies also validated that probiotic treatment and fecal microbial transplantation can achieve similar effect [228, 229].

## Cerebral vascular diseases

Cerebrovascular diseases are a variety of circulatory diseases that affect cerebral circulation and, thus, cause brain damage. The most common presentations of cerebrovascular disease are ischemic stroke, hemorrhagic stroke, and intracranial arteriovenous malformation (AVM). The GI microflora and infection have been indicated to impact the host immune system and ischemic stroke processes. Infection and inflammation at plaque lesions along with imbalanced carnitine, cholesterol and fat metabolism by intestinal microflora ultimately contributed to atherosclerosis.

### Atherosclerosis

Fernandes et al. revealed that oral *Streptococcus mutans* is ubiquitous in the atherosclerotic plaques of patients with vascular disease [230]. Apfalter et al. identified traces of *Chlamydia pneumoniae* in carotid artery atherosclerosis using a nested PCR-based approach [231]. Mitra et al.’s metagenomic study of carotid atherosclerosis plaques showed that 2–16% of the sequencing reads from plaque tissue came from bacteria, including *Lactobacillus rhamnosus* and *Neisseria polysaccharea*. The bacterial content is higher in patients with ischemic symptoms. *Acidovorax* spp. and *H. pylori* cells were also detected by fluorescence in situ hybridization (FISH) in atherosclerotic tissue [232]. Karlsson et al. sequenced the gut metagenomes of patients with symptomatic atherosclerosis and healthy subjects. The genus *Collinsella* was observed to be enriched in carotid stenotic patients, whereas *Roseburia* and *Eubacterium* were more abundant in healthy controls [233].The gut microbiota has been suggested to transform dietary choline, lecithin, or carnitine into TMAO and, thus, cause vascular atherosclerosis by affecting lipid and hormonal homeostasis [89, 234]. The carnitine-butyrobetaine-trimethylamine-*N*-oxide pathway has been found to be associated with carotid atherosclerosis [235]. Stimulation of monocyte by LPS can be a critical step of the formation of foam cell in atherosclerotic plaque by either inducing inflammation or triggering LDL uptake [236, 237]. A series of probiotic-based therapy attempts have been in progress, including human trials using *L. acidophilus* 145, *B. longum* 913 [238], *L. acidophilus* and *B. bifidum* [239] to restore HDL/LDL ratio, and mouse experiments using *L. plantarum ZDY04* against TMAO [240].

### Stroke

Benakis et al. showed that infarction-induced ischemic brain injury could be reduced by antibiotic administration. The antibiotic-induced alterations in the gut microbiota could increase regulatory T cells and decrease IL-17+ γδ T cells, which suppress the trafficking of effector T cells after stroke. Interestingly, the gut microbiota-associated infarction model can be transmitted and reproduced by fecal transplantation [241]. Systemic exposure to *Porphyromonas gingivalis* has been linked to an increased risk of ischemic stroke [242]. Kawato et al. revealed that continual venous injection of Gram-negative bacteria might accelerate stroke in SHRSP (spontaneously hypertensive stroke-prone) rats, probably due to LPS-induced oxidative stress responses [243]. Zhu et al. discovered that TMAO is associated with platelet hyperactivity and thrombosis risk [90]. Their following study validated in a short-term cohort that an omnivorous diet could generate a higher TMAO content in blood than was induced by a vegan diet, which was more likely to promote the formation of blood clots. Their study suggested that the use of low doses of aspirin was helpful to alleviate platelet aggregation caused by TMAO [244]. Yin et al. revealed that opportunistic gut-derived pathogens such as *Enterobacter*, *Megasphaera*, *Oscillibacter*, and *Desulfovibrio* were enriched in patients experiencing stroke and transient ischemic attack, whereas beneficial genera including *Bacteroides*, *Prevotella*, and *Faecalibacterium* were less abundant. Paradoxically, Yin et al. showed that TMAO levels were lower in patients than in controls [245].

### Arteriovenous malformation

There is no direct causal relationship between the microbiota and AVM onset in humans, while animal models have implied a pivotal role of the microflora in AVM pathophysiology and pathogenesis. Shikata et al. used antibiotics to deplete the gut microbiota in an aneurysm mouse model, and aneurysm induction was drastically reduced due to less macrophage infiltration and cytokine secretion [246]. Cerebral cavernous malformation (CCM) is a common type of vascular malformation resulting in hemorrhagic stroke and seizure [247]. Tang et al. showed that stimulation of TLR4 by bacteria or LPS accelerates CCM formation, whereas the use of germ-free mice and antibiotics use in mice can prevent CCM. The gut microbiome and endothelial TLR4 act as critical stimulants, suggesting their potential use as novel strategies in CCM therapy [248]. Interestingly, Winek et al.’s paradoxical results indicated that a broad-spectrum antibiotic-treated gut microbiota worsens inflammation and stroke in a murine model [249]. However, Evangelo Boumis et al. found that consumption of probiotics such as *Lactobacillus rhamnosus* may impose a serious risk of infection in patients with special susceptibility factors, and antibiotic prevention should be considered [250]. These findings implied that specific microbiota and antibiotic use result in different outcomes.

## Conclusions and perspectives

It is now gradually being accepted that the gut microbiota is important in both the maintenance of the intestinal flora and brain physiology. Through the immune system, endocrine system, and bacterial metabolites, the gut microbiota regulates neurotransmission and vascular barriers, which in turn alter host neuropsychological function, cognition, and cerebral vascular physiology. Research and development of potential therapeutic targets are also in progresses. The brain diseases, alteration of gut microbiota, evidenced mechanisms, and potential probiotic-based therapeutics are summarized in Table 2. However, there are still challenges in terms of the experimental design, subjects, models, analytical approach, pipeline, and quality control protocols used in metabolomics studies.

**Table 2:** The brain disorders, alteration of gut microbiota, pathological and molecular signature, and potential probiotic-based therapeutics

Brain disorders	Dysbiosis of microbiota	Pathological and molecular signature	Potential probiotic-based therapeutics
Stress and depression	Increase of *Faecalibacterium, Alistipes, Ruminococcus* [112], *Campylobacter jejuni* [143] and *Firmicutes*; decrease of *Bacteroidetes* [251]	Activation and releasing of HPA [121],VFA [112, 141], serotonin 1A receptor [122]; reduced expression of BDNF [122], GABA receptor [119]	*Bifidobacterium infantis* [121], *Lactobacillus rhamnosus* [119], *Lactobacillus helveticus*, *Bifidobacterium longum* [142], and other *Bifidobacterium* strains [143]
Pain and migraine	Increase of *H.pylor*i [162]; dysbiosis [163]	Dysfunction of CB2 and TLR7 [160]; inflammatory hypernociception [161]; TNF-α induced chronic migrain e[163]	*Lactobacillus*, *B. infantis* 35624 and *Lactobacillus farciminis* [165, 166], *Lactobacillus salivarius* UCC433 1[167]
Autism spectrum disorders	Decreases of *Faecalibacterium* species [150], *Bifidobacteria* species, *Akkermansia muciniphila*; increase of *Lactobacillus*, *Bacteroides, Prevotella*, and *Alistipes* [145, 151, 152]; alteration of *Fusobacteria*, *Verrucomicrobia* and *Firmicutes/Bacteroides* ratio [11]	Disrupted intestinal epithelial permeability [114], mucosal inflammation [148, 149], reduction of SCFA synthesis [150], mucosal inflammation [148, 149]	*Bacteroides fragilis* [114], *Lactobacillus* species and *Bifidobacterium* species [119, 155]
Parkinson’s disease	Increase of *H.pylori* [174], *E.coli* [179], *Ralstonia*, *Oscillospira* and *Bacteroides* [180]; decrease of *Prevotellaceae* [181], *Blautia*, *Coprococcus*, and *Roseburia* [180]	Higher frequency of α-synuclein detection [175], dopaminergic neuronal loss [177], bowel inflammation and change of permeability [178, 180]	Multiple probiotics strains (*Streptococcus salivarius subsp thermophilus, Enterococcus faecium, Lactobacillus rhamnosus GG, Lactobacillus acidophilus, Lactobacillus plantarum, Lactobacillus paracasei, Lactobacillus delbrueckii subsp bulgaricus*, and *Bifidobacterium*) [182]
Alzheimer’s disease	Chronic *H.pylori* infection [193, 194]; increase of *E.coli*, *Salmonella* spp, *Pseudomonas fluorescens*, *Klebsiella pneumonia*, *Staphylococcus aureus*, *Bacillus subtills*, *Streptomyces coelicolor* [190, 196, 197]; *Chlamydia pneumoniae* infection [199]	Increased levels of IL-1beta, IL-6, and TNF-alpha [186, 187]; extracellular amyloids such as CsgA, Aβ42, FapC, MccE492, phenol-soluble Modulins, TasA, Chaplins, and β-amyloid exhibit cerebral deposition [190, 192, 196, 197, 199]	*Lactobacilli* and *Bifidobacteria* [200, 203]; *Lactobacillus fermentum NS9* and *Lactobacillus helveticus NS8* [201, 202]
Amyotrophic lateral sclerosis	Reduced levels of butyrate-producing bacteria, including *Butyrivibrio fibrisolvens*, *Escherichia coli*, *Oscillibacter, Anaerostipes,* and *Lachnospira* [213]; increase of glucose-metabolizing *Dorea* [214]	Tetanus and botulinum toxins [210, 211]; “leaky gut” [212]; higher richness of OTUs [215]; reduction of butyrate [216]	*Lactobacillus* strains [217]
Multiple sclerosis	Increase of *Archaea, Psuedomonas, Haemophilus, Blautia*, *Dorea* [219], and *Fusobacteria* [225]; decrease of *Bacteroidetes phyla, Firmicutes phyla,* [218] *Parabacteroides, Adlercreutzia*, *Prevotella* [219], *Bacteroides* and *Clostridia* [218, 222]	BBB integrity disruption and astrocyte pathogenicity [222]; increasing proinflammatory cell infiltration and impaired Treg function [221]; “leaky gut” [219, 220]	*Bifidobacterium* and lactic acid-producing bacteria [223, 224]; *Bacteroidaceae, Faecalibacterium, Ruminococcus, Lactobacillaceae*, and *Clostridium* [226]
Atherosclerosis	*Lactobacillus rhamnosus* and *Neisseria polysaccharea, Acidovorax* spp. and *H. pylori* cells [232]; *Collinsella* [233]; *Roseburia* and *Eubacterium* [233]	Carnitine-butyrobetaine-trimethylamine-*N*-oxide pathway [235]; cause vascular atherosclerosis by affecting lipid and hormonal homeostasis [82, 222]; formation of foam cell by inflammation [236, 237]	*L. acidophilus* 145, *B. longum* 91 3[238], *L. acidophilus* and *B. bifidum* [239], *L. plantarum ZDY04* against TMAO [240]
Stroke	Increase of *Porphyromonas gingivalis* [242], Gram-negative bacteria [243], *Enterobacter*, *Megasphaera*, *Oscillibacter*; decrease of *Bacteroides*, *Prevotella* and *Faecalibacterium* [245]	Formation of blood clots and platelet aggregation [244]; decrease of regulatory T cells [241]	Antibiotic administration [241]
Arteriovenous malformation	Gram-negative bacteria [248]	Macrophage infiltration and cytokine secretion [246]; activate TLR4 [248]	Antibiotic and probiotics treatment are controversial [248–250]

Thus far, evidence has revealed clear associations between microbiota and host physiology, rather than demonstrating causal relationships. Since there are multiple confounding variables in human fecal experiments, larger sample-size studies are needed for metagenomic biomarker screening. Such studies are more reliable when exposure, demographics, diet, and socioeconomic factors are considered. However, even in population-based metagenomic analyses, the outcome variables are largely influenced by irreducible variables and confounders, while the composition of the microbiome can be explained by limited effects (10–20%) [252–254]. Another obstacle in current microbiota-based translational medicine is the mild and long-term of effects observed in terms of cognitive or psychological function. It might take months to years of probiotic and microbiota-based therapeutics to influence neuropsychiatric diseases, while the effect of the microbiota on host coagulation can be observed much more rapidly.

In addition to human cohort and cross-sectional studies, animal models provide essential evidence to explain how specific microbes affect the host. Animal models, especially murine models, have been widely adopted in pre-clinical experiments to validate the functions of specific microbial species. Early animal studies were established in germ-free animals to reveal associations between host physiology and the effect of the microbiota [255]. Specific microorganisms colonizing germ-free animals, probiotic use and fecal microbiota transplantation (FMT) approaches are widely used to elucidate the specific traits and functions of the microbiota. Antibiotic-treated animal models are alternatives for studying the microbial depletion effect on wild-type animals with mature immunity [20]. However, there are still many obstacles in the practice of translational medicine using conclusions about the microbiome from animal models, including issues related to genomic background, intestinal differences, dietary habits, and other life exposures. For example, although the human genome shares more than 85% of its genomic sequences with the mouse genome, the expression patterns and protein functions of these species are not exactly the same [256–259]. In terms of intestinal structure, mice present a relatively larger cecum and small intestine than humans. On the other hand, there are more circular folds (i.e., plicae circularis) in the human small intestine that increase the surface area, whereas the appearance of the mouse mucosa surface is smoother [259, 260]. Life experience is another issue that arises during data analysis, as humans and animals experience different life histories, exercise regimes, circadian cycles, social pressures, and especially food contents. Normally, the diet and chewing in animal experiments are nutritionally controlled and monotonous, while the composition of the human diet varies daily. These diet-related confounders may cause challenges during replication and translation of a model into clinical trials. However, quantitative cohort studies have provided stronger evidence than retrospective questionnaires in human studies, and smaller sample sizes are sometimes adequate to validate a hypothesis [244, 261].

The choice of microbiome analysis approaches and their technical stability are additional major challenges. The DNA extraction method, library preparation protocol, sequencer and analytic pipeline employed contribute to the observed biological variation even within a given specimen [262]. Recently, large-scale comparative studies identified the importance of sample homogenization, aliquoting and analytical pipelines in data processing [263, 264]. It is also worth noting that the results from 16S and WMS analyses are not fully replicable, since 16S sequencing largely depends on variable region selection, while WMS identifies and quantitates fragments from the entire genome of all organisms in the sample. Therefore, each protocol exhibits a preference for specific taxonomic assignments, which might cause further misinterpretations [265–268]. Furthermore, most 16S sequencing protocols obtain phylum- or genus-level resolution of taxa, whereas the WMS protocol can provide species or even strain-level information [24, 269].

Although far from being perfect, the gut microbiota-based therapy is a promising potential approach to be used in future therapies for brain diseases. Doctors and scientists are ready to think outside the pillbox in accord with the suggestion of Hippocrates (400 BC) to “Let food be thy medicine and medicine be thy food” [270]. Scientists are also pushing the frontier of the application of the microbiota in the diagnosis, treatment, and prognosis of brain diseases. The robust data produced from MWAS, metabolomics, and multi-omics are hopefully forming a framework that will enable the integration of psychological care, cerebrovascular care, and gastroenterological care into therapies.

## Data Availability

Not applicable

## Abbreviations

4-EPS: 4-ethylphenylsulfate
AD: Alzheimer’s disease
AHR: Aryl hydrocarbon receptor
ALS: Amyotrophic lateral sclerosis
APP: Aβ precursor protein
ASDs: Autism spectrum disorders
AVM: Arteriovenous malformation
Aβ: Beta-amyloid
BBB: Blood–brain barrier
CCM: Cerebral cavernous malformation
CNS: Central nervous system
DASH: Dietary approach to stop hypertension
DMB: 3-dimethyl-1-butanol
EAE: Experimental autoimmune encephalomyelitis
FISH: Fluorescence in situ hybridization
FMT: Fecal microbiota transplantation
GABA: Gamma-aminobutyric acid
HFD: High-fat diet
HFHC: High-fat and high-cholesterol diet
HMP: Human Microbiome Project
HPA: Hypothalamus-pituitary-adrenal gland axis
I3S: Indoxyl-3-sulfate
IAld: Indole-3-aldehyde
IBD: Inflammatory bowel diseases
IBS: Irritable bowel syndrome
ILC3: Group 3 innate lymphoid cells
IPA: Indole-3-propionic acid
ITS: Internal transcribed spacer
LCFAs: Long-chain fatty acids
LPS: Lipopolysaccharides
LTA: Lipoteichoic acid
MeDi: Mediterranean diet
MetaHIT: Metagenomics of the human intestinal tract
MIND: Mediterranean-DASH Intervention for Neurodegenerative Delay
MMSE: Mini-mental state examination
MRI: Magnetic resonance imaging
MS: Multiple sclerosis
MWAS: Metagenome-wide association studies
NGS: Next-generation sequencing
NMRS: Nuclear magnetic resonance spectroscopy
NMS: Non-motor symptoms
PAMPs: Pathogen-associated molecular patterns
PD: Parkinson’s disease
PRRs: Pattern recognition receptors
rRNA: Ribosomal RNA
SCFAs: Short-chain fatty acids
SHRSP: Spontaneously hypertensive stroke-prone
SIBO: Small intestinal bacterial overgrowth
SSU: Small subunit
TLR: Toll-like receptor
TMA: Trimethylamine
TMAO: Trimethylamine oxide
TnAse: Tryptophanase
Tregs: Regulatory T cells
VFA: Volatile fatty acid

## References

[1] Madigan MT (2012). Brock biology of microorganisms.

[2] Shulman ST, Friedmann HC, Sims RH (2007). Theodor Escherich: the first pediatric infectious diseases physician?. Clinical infectious diseases : an official publication of the Infectious Diseases Society of America.

[3] Podolsky SH (2012). Metchnikoff and the microbiome. Lancet.

[4] Tirosh I, Izar B, Prakadan SM, Wadsworth MH, Treacy D, Trombetta JJ, Rotem A, Rodman C, Lian C, Murphy G (2016). Dissecting the multicellular ecosystem of metastatic melanoma by single-cell RNA-seq. Science.

[5] Tan TZ, Miow QH, Miki Y, Noda T, Mori S, Huang RY, Thiery JP (2014). Epithelial-mesenchymal transition spectrum quantification and its efficacy in deciphering survival and drug responses of cancer patients. EMBO molecular medicine.

[6] Qin J, Li Y, Cai Z, Li S, Zhu J, Zhang F, Liang S, Zhang W, Guan Y, Shen D (2012). A metagenome-wide association study of gut microbiota in type 2 diabetes. Nature.

[7] Forslund K, Hildebrand F, Nielsen T, Falony G, Le Chatelier E, Sunagawa S, Prifti E, Vieira-Silva S, Gudmundsdottir V, Pedersen HK (2015). Disentangling type 2 diabetes and metformin treatment signatures in the human gut microbiota. Nature.

[8] Manichanh C, Rigottier-Gois L, Bonnaud E, Gloux K, Pelletier E, Frangeul L, Nalin R, Jarrin C, Chardon P, Marteau P (2006). Reduced diversity of faecal microbiota in Crohn's disease revealed by a metagenomic approach. Gut.

[9] Carroll IM, Ringel-Kulka T, Chang YH, Packey CD, Sartor RB, Ringel Y, Keku TO (2011). Molecular analysis of the luminal- and mucosal-associated intestinal microbiota in diarrhea-predominant irritable bowel syndrome. American journal of physiology Gastrointestinal and liver physiology.

[10] Zeller G, Tap J, Voigt AY, Sunagawa S, Kultima JR, Costea PI, Amiot A, Bohm J, Brunetti F, Habermann N (2014). Potential of fecal microbiota for early-stage detection of colorectal cancer. Molecular systems biology.

[11] De Angelis M, Francavilla R, Piccolo M, De Giacomo A, Gobbetti M (2015). Autism spectrum disorders and intestinal microbiota. Gut microbes.

[12] Rosenfeld CS (2015). Microbiome disturbances and autism spectrum disorders. Drug metabolism and disposition: the biological fate of chemicals.

[13] Winek K, Dirnagl U, Meisel A (2016). The Gut Microbiome as Therapeutic target in central nervous system diseases: implications for stroke. Neurotherapeutics : the journal of the American Society for Experimental NeuroTherapeutics.

[14] Collins SM, Surette M, Bercik P (2012). The interplay between the intestinal microbiota and the brain. Nature reviews Microbiology.

[15] Neunlist M, Van Landeghem L, Mahe MM, Derkinderen P, Des Varannes SB, Rolli-Derkinderen M (2013). The digestive neuronal-glial-epithelial unit: a new actor in gut health and disease. Nature reviews Gastroenterology & Hepatology.

[16] Furness JB (2012). The enteric nervous system and neurogastroenterology. Nature Reviews Gastroenterology & Hepatology.

[17] Matteoli G, Boeckxstaens GE (2013). The vagal innervation of the gut and immune homeostasis. Gut.

[18] Wang Y, Kasper LH (2014). The role of microbiome in central nervous system disorders. Brain, behavior, and immunity.

[19] Petra AI, Panagiotidou S, Hatziagelaki E, Stewart JM, Conti P, Theoharides TC (2015). Gut-microbiota-brain axis and its effect on neuropsychiatric disorders with suspected immune dysregulation. Clinical therapeutics.

[20] Knight R, Vrbanac A, Taylor BC, Aksenov A, Callewaert C, Debelius J, Gonzalez A, Kosciolek T, McCall LI, McDonald D (2018). Best practices for analysing microbiomes. Nature reviews Microbiology.

[21] Woese CR, Fox GE (1977). Phylogenetic structure of the prokaryotic domain: the primary kingdoms. Proceedings of the National Academy of Sciences of the United States of America.

[22] Woese CR, Stackebrandt E, Macke TJ, Fox GE (1985). A phylogenetic definition of the major eubacterial taxa. Systematic and applied microbiology.

[23] Wilson KH, Blitchington RB (1996). Human colonic biota studied by ribosomal DNA sequence analysis. Appl Environ Microbiol.

[24] Wang Q, Garrity GM, Tiedje JM, Cole JR (2007). Naive Bayesian classifier for rapid assignment of rRNA sequences into the new bacterial taxonomy. Appl Environ Microbiol.

[25] Hillmann B, Al-Ghalith GA, Shields-Cutler RR, Zhu Q, Gohl DM, Beckman KB, Knight R, Knights D. Evaluating the information content of shallow shotgun metagenomics. mSystems. 2018;3(6). 10.1128/mSystems.00069-18 PMC6234283 30443602

[26] Johnson JS, Spakowicz DJ, Hong BY, Petersen LM, Demkowicz P, Chen L, Leopold SR, Hanson BM, Agresta HO, Gerstein M (2019). Evaluation of 16S rRNA gene sequencing for species and strain-level microbiome analysis. Nat Commun.

[27] Breitbart M, Salamon P, Andresen B, Mahaffy JM, Segall AM, Mead D, Azam F, Rohwer F (2002). Genomic analysis of uncultured marine viral communities. Proceedings of the National Academy of Sciences of the United States of America.

[28] Gillespie DE, Brady SF, Bettermann AD, Cianciotto NP, Liles MR, Rondon MR, Clardy J, Goodman RM, Handelsman J (2002). Isolation of antibiotics turbomycin A and B from a metagenomic library of soil microbial DNA. Applied and environmental microbiology.

[29] Tyson GW, Chapman J, Hugenholtz P, Allen EE, Ram RJ, Richardson PM, Solovyev VV, Rubin EM, Rokhsar DS, Banfield JF (2004). Community structure and metabolism through reconstruction of microbial genomes from the environment. Nature.

[30] Wang J, Jia H (2016). Metagenome-wide association studies: fine-mining the microbiome. Nature reviews Microbiology.

[31] Franzosa EA, Hsu T, Sirota-Madi A, Shafquat A, Abu-Ali G, Morgan XC, Huttenhower C (2015). Sequencing and beyond: integrating molecular 'omics' for microbial community profiling. Nature reviews Microbiology.

[32] Heyer R, Schallert K, Zoun R, Becher B, Saake G, Benndorf D (2017). Challenges and perspectives of metaproteomic data analysis. Journal of biotechnology.

[33] Griffin JL, Wang X, Stanley E (2015). Does our gut microbiome predict cardiovascular risk? A review of the evidence from metabolomics. Circulation Cardiovascular genetics.

[34] Smirnov KS, Maier TV, Walker A, Heinzmann SS, Forcisi S, Martinez I, Walter J, Schmitt-Kopplin P (2016). Challenges of metabolomics in human gut microbiota research. International journal of medical microbiology : IJMM.

[35] Arrieta MC, Finlay BB (2012). The commensal microbiota drives immune homeostasis. Front Immunol.

[36] Okada H, Kuhn C, Feillet H, Bach JF (2010). The 'hygiene hypothesis' for autoimmune and allergic diseases: an update. Clin Exp Immunol.

[37] Medzhitov R, Janeway C (2000). Innate immune recognition: mechanisms and pathways. Immunological reviews.

[38] Janeway CA (1989). Approaching the asymptote? Evolution and revolution in immunology. Cold Spring Harbor symposia on quantitative biology.

[39] Konig J, Wells J, Cani PD, Garcia-Rodenas CL, MacDonald T, Mercenier A, Whyte J, Troost F, Brummer RJ (2016). Human intestinal barrier function in health and disease. Clinical and translational gastroenterology.

[40] Wells JM, Brummer RJ, Derrien M, MacDonald TT, Troost F, Cani PD, Theodorou V, Dekker J, Meheust A, de Vos WM (2017). Homeostasis of the gut barrier and potential biomarkers. American journal of physiology Gastrointestinal and liver physiology.

[41] Okun MS (2012). Deep-brain stimulation for Parkinson's disease. The New England journal of medicine.

[42] Rolls A, Shechter R, London A, Ziv Y, Ronen A, Levy R, Schwartz M (2007). Toll-like receptors modulate adult hippocampal neurogenesis. Nature cell biology.

[43] Shechter R, Ronen A, Rolls A, London A, Bakalash S, Young MJ, Schwartz M (2008). Toll-like receptor 4 restricts retinal progenitor cell proliferation. The Journal of cell biology.

[44] Okun E, Griffioen KJ, Son TG, Lee JH, Roberts NJ, Mughal MR, Hutchison E, Cheng A, Arumugam TV, Lathia JD (2010). TLR2 activation inhibits embryonic neural progenitor cell proliferation. Journal of neurochemistry.

[45] Keohane A, Ryan S, Maloney E, Sullivan AM, Nolan YM (2010). Tumour necrosis factor-alpha impairs neuronal differentiation but not proliferation of hippocampal neural precursor cells: role of Hes1. Molecular and cellular neurosciences.

[46] Okun E, Barak B, Saada-Madar R, Rothman SM, Griffioen KJ, Roberts N, Castro K, Mughal MR, Pita MA, Stranahan AM (2012). Evidence for a developmental role for TLR4 in learning and memory. PloS one.

[47] Wang S, Zhang X, Zhai L, Sheng X, Zheng W, Chu H, Zhang G (2018). Atorvastatin attenuates cognitive deficits and neuroinflammation induced by Abeta1-42 involving modulation of TLR4/TRAF6/NF-kappaB pathway. Journal of molecular neuroscience : MN.

[48] Honda K, Littman DR (2016). The microbiota in adaptive immune homeostasis and disease. Nature.

[49] Atarashi K, Tanoue T, Shima T, Imaoka A, Kuwahara T, Momose Y, Cheng G, Yamasaki S, Saito T, Ohba Y (2011). Induction of colonic regulatory T cells by indigenous Clostridium species. Science.

[50] Smith PM, Howitt MR, Panikov N, Michaud M, Gallini CA, Bohlooly YM, Glickman JN, Garrett WS (2013). The microbial metabolites, short-chain fatty acids, regulate colonic Treg cell homeostasis. Science.

[51] Mazmanian SK, Liu CH, Tzianabos AO, Kasper DL (2005). An immunomodulatory molecule of symbiotic bacteria directs maturation of the host immune system. Cell.

[52] Hirota K, Turner JE, Villa M, Duarte JH, Demengeot J, Steinmetz OM, Stockinger B (2013). Plasticity of Th17 cells in Peyer's patches is responsible for the induction of T cell-dependent IgA responses. Nature immunology.

[53] Ivanov II, McKenzie BS, Zhou L, Tadokoro CE, Lepelley A, Lafaille JJ, Cua DJ, Littman DR (2006). The orphan nuclear receptor RORgammat directs the differentiation program of proinflammatory IL-17+ T helper cells. Cell.

[54] Ivanov II, Frutos Rde L, Manel N, Yoshinaga K, Rifkin DB, Sartor RB, Finlay BB, Littman DR (2008). Specific microbiota direct the differentiation of IL-17-producing T-helper cells in the mucosa of the small intestine. Cell host & microbe.

[55] Ishigame H, Kakuta S, Nagai T, Kadoki M, Nambu A, Komiyama Y, Fujikado N, Tanahashi Y, Akitsu A, Kotaki H (2009). Differential roles of interleukin-17A and -17F in host defense against mucoepithelial bacterial infection and allergic responses. Immunity.

[56] Horai R, Zarate-Blades CR, Dillenburg-Pilla P, Chen J, Kielczewski JL, Silver PB, Jittayasothorn Y, Chan CC, Yamane H, Honda K (2015). Microbiota-dependent activation of an autoreactive T cell receptor provokes autoimmunity in an immunologically privileged site. Immunity.

[57] Berer K, Mues M, Koutrolos M, Rasbi ZA, Boziki M, Johner C, Wekerle H, Krishnamoorthy G (2011). Commensal microbiota and myelin autoantigen cooperate to trigger autoimmune demyelination. Nature.

[58] McGeachy MJ, Chen Y, Tato CM, Laurence A, Joyce-Shaikh B, Blumenschein WM, McClanahan TK, O'Shea JJ, Cua DJ (2009). The interleukin 23 receptor is essential for the terminal differentiation of interleukin 17-producing effector T helper cells in vivo. Nature immunology.

[59] Coccia M, Harrison OJ, Schiering C, Asquith MJ, Becher B, Powrie F, Maloy KJ (2012). IL-1beta mediates chronic intestinal inflammation by promoting the accumulation of IL-17A secreting innate lymphoid cells and CD4(+) Th17 cells. The Journal of experimental medicine.

[60] Schulte-Herbruggen O, Quarcoo D, Meisel A, Meisel C (2009). Differential affection of intestinal immune cell populations after cerebral ischemia in mice. Neuroimmunomodulation.

[61] Diaz Heijtz R, Wang S, Anuar F, Qian Y, Bjorkholm B, Samuelsson A, Hibberd ML, Forssberg H, Pettersson S (2011). Normal gut microbiota modulates brain development and behavior. Proceedings of the National Academy of Sciences of the United States of America.

[62] Obermeier B, Daneman R, Ransohoff RM (2013). Development, maintenance and disruption of the blood-brain barrier. Nature medicine.

[63] Daneman R, Prat A (2015). The blood-brain barrier. Cold Spring Harbor perspectives in biology.

[64] Acharya NK, Levin EC, Clifford PM, Han M, Tourtellotte R, Chamberlain D, Pollaro M, Coretti NJ, Kosciuk MC, Nagele EP (2013). Diabetes and hypercholesterolemia increase blood-brain barrier permeability and brain amyloid deposition: beneficial effects of the LpPLA2 inhibitor darapladib. Journal of Alzheimer’s disease : JAD.

[65] Fiorentino M, Sapone A, Senger S, Camhi SS, Kadzielski SM, Buie TM, Kelly DL, Cascella N, Fasano A (2016). Blood-brain barrier and intestinal epithelial barrier alterations in autism spectrum disorders. Molecular autism.

[66] Holmqvist S, Chutna O, Bousset L, Aldrin-Kirk P, Li W, Bjorklund T, Wang ZY, Roybon L, Melki R, Li JY (2014). Direct evidence of Parkinson pathology spread from the gastrointestinal tract to the brain in rats. Acta neuropathologica.

[67] Braniste V, Al-Asmakh M, Kowal C, Anuar F, Abbaspour A, Toth M, Korecka A, Bakocevic N, Ng LG, Kundu P (2014). The gut microbiota influences blood-brain barrier permeability in mice. Science translational medicine.

[68] Lee SW, Kim WJ, Choi YK, Song HS, Son MJ, Gelman IH, Kim YJ, Kim KW (2003). SSeCKS regulates angiogenesis and tight junction formation in blood-brain barrier. Nature medicine.

[69] Spadoni I, Fornasa G, Rescigno M (2017). Organ-specific protection mediated by cooperation between vascular and epithelial barriers. Nature reviews Immunology.

[70] Luczynski Pauline, McVey Neufeld Karen-Anne, Oriach Clara Seira, Clarke Gerard, Dinan Timothy G., Cryan John F. (2016). Growing up in a Bubble: Using Germ-Free Animals to Assess the Influence of the Gut Microbiota on Brain and Behavior. International Journal of Neuropsychopharmacology.

[71] Bao CH, Liu P, Liu HR, Wu LY, Shi Y, Chen WF, Qin W, Lu Y, Zhang JY, Jin XM (2015). Alterations in brain grey matter structures in patients with Crohn's disease and their correlation with psychological distress. Journal of Crohn's & colitis.

[72] Mrakotsky Christine, Anand Rajsavi, Watson Christopher, Vu Catherine, Matos Alana, Friel Shelby, Rivkin Michael, Snapper Scott (2016). O-018 New Evidence for Structural Brain Differences in Pediatric Crohnʼs Disease. Inflammatory Bowel Diseases.

[73] Fernandez-Real JM, Serino M, Blasco G, Puig J, Daunis-i-Estadella J, Ricart W, Burcelin R, Fernandez-Aranda F, Portero-Otin M (2015). Gut microbiota interacts with brain microstructure and function. The Journal of clinical endocrinology and metabolism.

[74] Vipperla K, O'Keefe SJ (2012). The microbiota and its metabolites in colonic mucosal health and cancer risk. Nutrition in clinical practice : official publication of the American Society for Parenteral and Enteral Nutrition.

[75] Luna RA, Foster JA (2015). Gut brain axis: diet microbiota interactions and implications for modulation of anxiety and depression. Current opinion in biotechnology.

[76] Evrensel A, Ceylan ME (2015). The gut-brain axis: the missing link in depression. Clinical psychopharmacology and neuroscience : the official scientific journal of the Korean College of Neuropsychopharmacology.

[77] Pistollato F, Iglesias RC, Ruiz R, Aparicio S, Crespo J, Lopez LD, Manna PP, Giampieri F, Battino M (2018). Nutritional patterns associated with the maintenance of neurocognitive functions and the risk of dementia and Alzheimer's disease: a focus on human studies. Pharmacological research.

[78] Dominguez LJ, Barbagallo M, Munoz-Garcia M, Godos J, Martinez-Gonzalez MA (2019). Dietary patterns and cognitive decline: key features for prevention. Current pharmaceutical design.

[79] Tangney CC, Li H, Wang Y, Barnes L, Schneider JA, Bennett DA, Morris MC (2014). Relation of DASH- and Mediterranean-like dietary patterns to cognitive decline in older persons. Neurology.

[80] Harrison CA, Taren D (2018). How poverty affects diet to shape the microbiota and chronic disease. Nature reviews Immunology.

[81] Hildebrandt MA, Hoffmann C, Sherrill-Mix SA, Keilbaugh SA, Hamady M, Chen YY, Knight R, Ahima RS, Bushman F, Wu GD (2009). High-fat diet determines the composition of the murine gut microbiome independently of obesity. Gastroenterology.

[82] Perry RJ, Peng L, Barry NA, Cline GW, Zhang D, Cardone RL, Petersen KF, Kibbey RG, Goodman AL, Shulman GI (2016). Acetate mediates a microbiome-brain-beta-cell axis to promote metabolic syndrome. Nature.

[83] Turnbaugh PJ, Ridaura VK, Faith JJ, Rey FE, Knight R, Gordon JI (2009). The effect of diet on the human gut microbiome: a metagenomic analysis in humanized gnotobiotic mice. Sci Transl Med.

[84] Li Q, Lauber CL, Czarnecki-Maulden G, Pan Y, Hannah SS. Effects of the dietary protein and carbohydrate ratio on gut microbiomes in dogs of different body conditions. MBio. 2017:8(1). 10.1128/mBio.01703-16 PMC5263242 28119466

[85] Wu GD, Chen J, Hoffmann C, Bittinger K, Chen YY, Keilbaugh SA, Bewtra M, Knights D, Walters WA, Knight R (2011). Linking long-term dietary patterns with gut microbial enterotypes. Science.

[86] De Filippis F, Pellegrini N, Vannini L, Jeffery IB, La Storia A, Laghi L, Serrazanetti DI, Di Cagno R, Ferrocino I, Lazzi C (2016). High-level adherence to a Mediterranean diet beneficially impacts the gut microbiota and associated metabolome. Gut.

[87] Li JM, Yu R, Zhang LP, Wen SY, Wang SJ, Zhang XY, Xu Q, Kong LD (2019). Dietary fructose-induced gut dysbiosis promotes mouse hippocampal neuroinflammation: a benefit of short-chain fatty acids. Microbiome.

[88] Wang Z, Klipfell E, Bennett BJ, Koeth R, Levison BS, Dugar B, Feldstein AE, Britt EB, Fu X, Chung YM (2011). Gut flora metabolism of phosphatidylcholine promotes cardiovascular disease. Nature.

[89] Koeth RA, Wang Z, Levison BS, Buffa JA, Org E, Sheehy BT, Britt EB, Fu X, Wu Y, Li L (2013). Intestinal microbiota metabolism of L-carnitine, a nutrient in red meat, promotes atherosclerosis. Nature medicine.

[90] Zhu W, Gregory JC, Org E, Buffa JA, Gupta N, Wang Z, Li L, Fu X, Wu Y, Mehrabian M (2016). Gut microbial metabolite TMAO ehances platelet hyperreactivity and thrombosis risk. Cell.

[91] Koren O, Spor A, Felin J, Fak F, Stombaugh J, Tremaroli V, Behre CJ, Knight R, Fagerberg B, Ley RE (2011). Human oral, gut, and plaque microbiota in patients with atherosclerosis. Proceedings of the National Academy of Sciences of the United States of America.

[92] Tang WH, Wang Z, Levison BS, Koeth RA, Britt EB, Fu X, Wu Y, Hazen SL (2013). Intestinal microbial metabolism of phosphatidylcholine and cardiovascular risk. The New England journal of medicine.

[93] Wang Z, Roberts AB, Buffa JA, Levison BS, Zhu W, Org E, Gu X, Huang Y, Zamanian-Daryoush M, Culley MK (2015). Non-lethal Inhibition of gut microbial trimethylamine production for the treatment of atherosclerosis. Cell.

[94] Jie Z, Xia H, Zhong SL, Feng Q, Li S, Liang S, Zhong H, Liu Z, Gao Y, Zhao H (2017). The gut microbiome in atherosclerotic cardiovascular disease. Nature communications.

[95] Olson CA, Vuong HE, Yano JM, Liang QY, Nusbaum DJ, Hsiao EY (2018). The gut microbiota mediates the anti-seizure effects of the ketogenic diet. Cell.

[96] Kelder T, Stroeve JH, Bijlsma S, Radonjic M, Roeselers G (2014). Correlation network analysis reveals relationships between diet-induced changes in human gut microbiota and metabolic health. Nutrition & diabetes.

[97] He K, Hu Y, Ma H, Zou Z, Xiao Y, Yang Y, Feng M, Li X, Ye X (2016). Rhizoma Coptidis alkaloids alleviate hyperlipidemia in B6 mice by modulating gut microbiota and bile acid pathways. Biochimica et biophysica acta.

[98] Bourassa MW, Alim I, Bultman SJ, Ratan RR (2016). Butyrate, neuroepigenetics and the gut microbiome: can a high fiber diet improve brain health?. Neuroscience letters.

[99] Topping DL, Clifton PM (2001). Short-chain fatty acids and human colonic function: roles of resistant starch and nonstarch polysaccharides. Physiological reviews.

[100] Haghikia A, Jorg S, Duscha A, Berg J, Manzel A, Waschbisch A, Hammer A, Lee DH, May C, Wilck N (2016). Dietary fatty acids directly impact central nervous system autoimmunity via the small intestine. Immunity.

[101] Schirmer M, Smeekens SP, Vlamakis H, Jaeger M, Oosting M, Franzosa EA, Ter Horst R, Jansen T, Jacobs L, Bonder MJ (2016). Linking the human gut microbiome to inflammatory cytokine production capacity. Cell.

[102] Duncan SH, Holtrop G, Lobley GE, Calder AG, Stewart CS, Flint HJ (2004). Contribution of acetate to butyrate formation by human faecal bacteria. The British journal of nutrition.

[103] Tan J, McKenzie C, Potamitis M, Thorburn AN, Mackay CR, Macia L (2014). The role of short-chain fatty acids in health and disease. Advances in immunology.

[104] Macfarlane S, Macfarlane GT (2003). Regulation of short-chain fatty acid production. The Proceedings of the Nutrition Society.

[105] Maslowski KM, Vieira AT, Ng A, Kranich J, Sierro F, Yu D, Schilter HC, Rolph MS, Mackay F, Artis D (2009). Regulation of inflammatory responses by gut microbiota and chemoattractant receptor GPR43. Nature.

[106] Kasubuchi M, Hasegawa S, Hiramatsu T, Ichimura A, Kimura I (2015). Dietary gut microbial metabolites, short-chain fatty acids, and host metabolic regulation. Nutrients.

[107] Krautkramer KA, Kreznar JH, Romano KA, Vivas EI, Barrett-Wilt GA, Rabaglia ME, Keller MP, Attie AD, Rey FE, Denu JM (2016). Diet-microbiota interactions mediate global epigenetic programming in multiple host tissues. Molecular cell.

[108] Rothhammer V, Borucki DM, Tjon EC, Takenaka MC, Chao CC, Ardura-Fabregat A, de Lima KA, Gutierrez-Vazquez C, Hewson P, Staszewski O (2018). Microglial control of astrocytes in response to microbial metabolites. Nature.

[109] Rothhammer V, Mascanfroni ID, Bunse L, Takenaka MC, Kenison JE, Mayo L, Chao CC, Patel B, Yan R, Blain M (2016). Type I interferons and microbial metabolites of tryptophan modulate astrocyte activity and central nervous system inflammation via the aryl hydrocarbon receptor. Nature medicine.

[110] Schroeder JC, Dinatale BC, Murray IA, Flaveny CA, Liu Q, Laurenzana EM, Lin JM, Strom SC, Omiecinski CJ, Amin S (2010). The uremic toxin 3-indoxyl sulfate is a potent endogenous agonist for the human aryl hydrocarbon receptor. Biochemistry.

[111] Jaglin M, Rhimi M, Philippe C, Pons N, Bruneau A, Goustard B, Dauge V, Maguin E, Naudon L, Rabot S (2018). Indole, a signaling molecule droduced by the gut microbiota, negatively impacts emotional behaviors in rats. Frontiers in neuroscience.

[112] Szczesniak O, Hestad KA, Hanssen JF, Rudi K (2016). Isovaleric acid in stool correlates with human depression. Nutritional neuroscience.

[113] Wu GD, Compher C, Chen EZ, Smith SA, Shah RD, Bittinger K, Chehoud C, Albenberg LG, Nessel L, Gilroy E (2016). Comparative metabolomics in vegans and omnivores reveal constraints on diet-dependent gut microbiota metabolite production. Gut.

[114] Hsiao EY, McBride SW, Hsien S, Sharon G, Hyde ER, McCue T, Codelli JA, Chow J, Reisman SE, Petrosino JF (2013). Microbiota modulate behavioral and physiological abnormalities associated with neurodevelopmental disorders. Cell.

[115] Miles C, Green R, Hines M (2006). Estrogen treatment effects on cognition, memory and mood in male-to-female transsexuals. Hormones and behavior.

[116] Keefer L (2018). Behavioural medicine and gastrointestinal disorders: the promise of positive psychology. Nature reviews Gastroenterology & hepatology.

[117] Clarke G, Grenham S, Scully P, Fitzgerald P, Moloney RD, Shanahan F, Dinan TG, Cryan JF (2013). The microbiome-gut-brain axis during early life regulates the hippocampal serotonergic system in a sex-dependent manner. Molecular psychiatry.

[118] Messaoudi M, Lalonde R, Violle N, Javelot H, Desor D, Nejdi A, Bisson JF, Rougeot C, Pichelin M, Cazaubiel M (2011). Assessment of psychotropic-like properties of a probiotic formulation (Lactobacillus helveticus R0052 and Bifidobacterium longum R0175) in rats and human subjects. The British journal of nutrition.

[119] Bravo JA, Forsythe P, Chew MV, Escaravage E, Savignac HM, Dinan TG, Bienenstock J, Cryan JF (2011). Ingestion of Lactobacillus strain regulates emotional behavior and central GABA receptor expression in a mouse via the vagus nerve. Proceedings of the National Academy of Sciences of the United States of America.

[120] Janik R, Thomason LAM, Stanisz AM, Forsythe P, Bienenstock J, Stanisz GJ (2016). Magnetic resonance spectroscopy reveals oral Lactobacillus promotion of increases in brain GABA, N-acetyl aspartate and glutamate. NeuroImage.

[121] Sudo N, Chida Y, Aiba Y, Sonoda J, Oyama N, Yu XN, Kubo C, Koga Y (2004). Postnatal microbial colonization programs the hypothalamic-pituitary-adrenal system for stress response in mice. The Journal of physiology.

[122] Neufeld KM, Kang N, Bienenstock J, Foster JA (2011). Reduced anxiety-like behavior and central neurochemical change in germ-free mice. Neurogastroenterology and motility : the official journal of the European Gastrointestinal Motility Society.

[123] Bercik P, Verdu EF, Foster JA, Macri J, Potter M, Huang X, Malinowski P, Jackson W, Blennerhassett P, Neufeld KA (2010). Chronic gastrointestinal inflammation induces anxiety-like behavior and alters central nervous system biochemistry in mice. Gastroenterology.

[124] Bercik P, Denou E, Collins J, Jackson W, Lu J, Jury J, Deng Y, Blennerhassett P, Macri J, McCoy KD (2011). The intestinal microbiota affect central levels of brain-derived neurotropic factor and behavior in mice. Gastroenterology.

[125] Crumeyrolle-Arias M, Jaglin M, Bruneau A, Vancassel S, Cardona A, Dauge V, Naudon L, Rabot S (2014). Absence of the gut microbiota enhances anxiety-like behavior and neuroendocrine response to acute stress in rats. Psychoneuroendocrinology.

[126] Desbonnet L, Clarke G, Shanahan F, Dinan TG, Cryan JF (2014). Microbiota is essential for social development in the mouse. Molecular psychiatry.

[127] Degroote S, Hunting DJ, Baccarelli AA, Takser L (2016). Maternal gut and fetal brain connection: increased anxiety and reduced social interactions in Wistar rat offspring following peri-conceptional antibiotic exposure. Progress in neuro-psychopharmacology & biological psychiatry.

[128] Frohlich EE, Farzi A, Mayerhofer R, Reichmann F, Jacan A, Wagner B, Zinser E, Bordag N, Magnes C, Frohlich E (2016). Cognitive impairment by antibiotic-induced gut dysbiosis: analysis of gut microbiota-brain communication. Brain, behavior, and immunity.

[129] Gareau MG, Wine E, Rodrigues DM, Cho JH, Whary MT, Philpott DJ, Macqueen G, Sherman PM (2011). Bacterial infection causes stress-induced memory dysfunction in mice. Gut.

[130] Mohle L, Mattei D, Heimesaat MM, Bereswill S, Fischer A, Alutis M, French T, Hambardzumyan D, Matzinger P, Dunay IR (2016). Ly6C(hi) monocytes provide a link between antibiotic-induced changes in gut microbiota and adult hippocampal neurogenesis. Cell reports.

[131] Lyte M, Li W, Opitz N, Gaykema RP, Goehler LE (2006). Induction of anxiety-like behavior in mice during the initial stages of infection with the agent of murine colonic hyperplasia Citrobacter rodentium. Physiology & behavior.

[132] Knight R, Jansson J, Field D, Fierer N, Desai N, Fuhrman JA, Hugenholtz P, van der Lelie D, Meyer F, Stevens R (2012). Unlocking the potential of metagenomics through replicated experimental design. Nature biotechnology.

[133] Blaser MJ, Dominguez-Bello MG (2016). The human microbiome before birth. Cell host & microbe.

[134] Kostic AD, Gevers D, Siljander H, Vatanen T, Hyotylainen T, Hamalainen AM, Peet A, Tillmann V, Poho P, Mattila I (2015). The dynamics of the human infant gut microbiome in development and in progression toward type 1 diabetes. Cell host & microbe.

[135] Williamson LL, Sholar PW, Mistry RS, Smith SH, Bilbo SD (2011). Microglia and memory: modulation by early-life infection. The Journal of neuroscience : the official journal of the Society for Neuroscience.

[136] Andre C, Dinel AL, Ferreira G, Laye S, Castanon N (2014). Diet-induced obesity progressively alters cognition, anxiety-like behavior and lipopolysaccharide-induced depressive-like behavior: focus on brain indoleamine 2,3-dioxygenase activation. Brain, behavior, and immunity.

[137] Magnusson KR, Hauck L, Jeffrey BM, Elias V, Humphrey A, Nath R, Perrone A, Bermudez LE (2015). Relationships between diet-related changes in the gut microbiome and cognitive flexibility. Neuroscience.

[138] O'Mahony SM, Marchesi JR, Scully P, Codling C, Ceolho AM, Quigley EM, Cryan JF, Dinan TG (2009). Early life stress alters behavior, immunity, and microbiota in rats: implications for irritable bowel syndrome and psychiatric illnesses. Biological psychiatry.

[139] Bangsgaard Bendtsen KM, Krych L, Sorensen DB, Pang W, Nielsen DS, Josefsen K, Hansen LH, Sorensen SJ, Hansen AK (2012). Gut microbiota composition is correlated to grid floor induced stress and behavior in the BALB/c mouse. PloS one.

[140] Banerjee A, Sarkhel S, Sarkar R, Dhali GK (2017). Anxiety and depression in irritable bowel syndrome. Indian journal of psychological medicine.

[141] Owens M, Herbert J, Jones PB, Sahakian BJ, Wilkinson PO, Dunn VJ, Croudace TJ, Goodyer IM (2014). Elevated morning cortisol is a stratified population-level biomarker for major depression in boys only with high depressive symptoms. Proceedings of the National Academy of Sciences of the United States of America.

[142] Ait-Belgnaoui A, Colom A, Braniste V, Ramalho L, Marrot A, Cartier C, Houdeau E, Theodorou V, Tompkins T (2014). Probiotic gut effect prevents the chronic psychological stress-induced brain activity abnormality in mice. Neurogastroenterology and motility : the official journal of the European Gastrointestinal Motility Society.

[143] de Theije CG, Wopereis H, Ramadan M, van Eijndthoven T, Lambert J, Knol J, Garssen J, Kraneveld AD, Oozeer R (2014). Altered gut microbiota and activity in a murine model of autism spectrum disorders. Brain, behavior, and immunity.

[144] Curran LK, Newschaffer CJ, Lee LC, Crawford SO, Johnston MV, Zimmerman AW (2007). Behaviors associated with fever in children with autism spectrum disorders. Pediatrics.

[145] Sandler RH, Finegold SM, Bolte ER, Buchanan CP, Maxwell AP, Vaisanen ML, Nelson MN, Wexler HM (2000). Short-term benefit from oral vancomycin treatment of regressive-onset autism. Journal of child neurology.

[146] Critchfield JW, van Hemert S, Ash M, Mulder L, Ashwood P (2011). The potential role of probiotics in the management of childhood autism spectrum disorders. Gastroenterology research and practice.

[147] Klukowski M, Wasilewska J, Lebensztejn D (2015). Sleep and gastrointestinal disturbances in autism spectrum disorder in children. Developmental period medicine.

[148] Chaidez V, Hansen RL, Hertz-Picciotto I (2014). Gastrointestinal problems in children with autism, developmental delays or typical development. Journal of autism and developmental disorders.

[149] Horvath K, Perman JA (2002). Autistic disorder and gastrointestinal disease. Current opinion in pediatrics.

[150] De Angelis M, Piccolo M, Vannini L, Siragusa S, De Giacomo A, Serrazzanetti DI, Cristofori F, Guerzoni ME, Gobbetti M, Francavilla R (2013). Fecal microbiota and metabolome of children with autism and pervasive developmental disorder not otherwise specified. PloS one.

[151] Adams JB, Johansen LJ, Powell LD, Quig D, Rubin RA (2011). Gastrointestinal flora and gastrointestinal status in children with autism--comparisons to typical children and correlation with autism severity. BMC gastroenterology.

[152] Wang L, Christophersen CT, Sorich MJ, Gerber JP, Angley MT, Conlon MA (2011). Low relative abundances of the mucolytic bacterium Akkermansia muciniphila and Bifidobacterium spp. in feces of children with autism. Applied and environmental microbiology.

[153] Finegold SM, Dowd SE, Gontcharova V, Liu C, Henley KE, Wolcott RD, Youn E, Summanen PH, Granpeesheh D, Dixon D (2010). Pyrosequencing study of fecal microflora of autistic and control children. Anaerobe.

[154] Parracho HM, Bingham MO, Gibson GR, McCartney AL (2005). Differences between the gut microflora of children with autistic spectrum disorders and that of healthy children. Journal of medical microbiology.

[155] Saulnier DM, Ringel Y, Heyman MB, Foster JA, Bercik P, Shulman RJ, Versalovic J, Verdu EF, Dinan TG, Hecht G (2013). The intestinal microbiome, probiotics and prebiotics in neurogastroenterology. Gut microbes.

[156] Theodorou V, Ait Belgnaoui A, Agostini S, Eutamene H (2014). Effect of commensals and probiotics on visceral sensitivity and pain in irritable bowel syndrome. Gut microbes.

[157] Sachdev AH, Pimentel M (2013). Gastrointestinal bacterial overgrowth: pathogenesis and clinical significance. Therapeutic advances in chronic disease.

[158] Faraji F, Zarinfar N, Zanjani AT, Morteza A (2012). The effect of Helicobacter pylori eradication on migraine: a randomized, double blind, controlled trial. Pain physician.

[159] Smilowicz A (2013). An osteopathic approach to gastrointestinal disease: somatic clues for diagnosis and clinical challenges associated with Helicobacter pylori antibiotic resistance. The Journal of the American Osteopathic Association.

[160] Aguilera M, Cerda-Cuellar M, Martinez V (2015). Antibiotic-induced dysbiosis alters host-bacterial interactions and leads to colonic sensory and motor changes in mice. Gut microbes.

[161] Amaral FA, Sachs D, Costa VV, Fagundes CT, Cisalpino D, Cunha TM, Ferreira SH, Cunha FQ, Silva TA, Nicoli JR (2008). Commensal microbiota is fundamental for the development of inflammatory pain. Proceedings of the National Academy of Sciences of the United States of America.

[162] Su J, Zhou XY, Zhang GX (2014). Association between Helicobacter pylori infection and migraine: a meta-analysis. World journal of gastroenterology.

[163] Tang Y, Liu S, Shu H, Yanagisawa L, Tao F. Gut microbiota dysbiosis enhances migraine-like pain via TNFalpha upregulation. Molecular neurobiology. 2019. 10.1007/s12035-019-01721-7 PMC6980505 31378003

[164] Rousseaux C, Thuru X, Gelot A, Barnich N, Neut C, Dubuquoy L, Dubuquoy C, Merour E, Geboes K, Chamaillard M (2007). Lactobacillus acidophilus modulates intestinal pain and induces opioid and cannabinoid receptors. Nature medicine.

[165] McKernan DP, Fitzgerald P, Dinan TG, Cryan JF (2010). The probiotic Bifidobacterium infantis 35624 displays visceral antinociceptive effects in the rat. Neurogastroenterology and motility : the official journal of the European Gastrointestinal Motility Society.

[166] Ait-Belgnaoui A, Eutamene H, Houdeau E, Bueno L, Fioramonti J, Theodorou V (2009). Lactobacillus farciminis treatment attenuates stress-induced overexpression of Fos protein in spinal and supraspinal sites after colorectal distension in rats. Neurogastroenterology and motility : the official journal of the European Gastrointestinal Motility Society.

[167] O'Mahony L, McCarthy J, Kelly P, Hurley G, Luo F, Chen K, O'Sullivan GC, Kiely B, Collins JK, Shanahan F (2005). Lactobacillus and bifidobacterium in irritable bowel syndrome: symptom responses and relationship to cytokine profiles. Gastroenterology.

[168] Pellegrini C, Antonioli L, Colucci R, Blandizzi C, Fornai M (2018). Interplay among gut microbiota, intestinal mucosal barrier and enteric neuro-immune system: a common path to neurodegenerative diseases?. Acta neuropathologica.

[169] Spielman LJ, Gibson DL, Klegeris A (2018). Unhealthy gut, unhealthy brain: the role of the intestinal microbiota in neurodegenerative diseases. Neurochemistry international.

[170] Tilvis RS, Kahonen-Vare MH, Jolkkonen J, Valvanne J, Pitkala KH, Strandberg TE (2004). Predictors of cognitive decline and mortality of aged people over a 10-year period. The journals of gerontology Series A, Biological sciences and medical sciences.

[171] Serres S, Anthony DC, Jiang Y, Broom KA, Campbell SJ, Tyler DJ, van Kasteren SI, Davis BG, Sibson NR (2009). Systemic inflammatory response reactivates immune-mediated lesions in rat brain. The Journal of neuroscience : the official journal of the Society for Neuroscience.

[172] Reeve A, Simcox E, Turnbull D (2014). Ageing and Parkinson's disease: why is advancing age the biggest risk factor?. Ageing research reviews.

[173] Felice VD, Quigley EM, Sullivan AM, O'Keeffe GW, O'Mahony SM (2016). Microbiota-gut-brain signalling in Parkinson's disease: implications for non-motor symptoms. Parkinsonism & related disorders.

[174] Fasano A, Visanji NP, Liu LW, Lang AE, Pfeiffer RF (2015). Gastrointestinal dysfunction in Parkinson's disease. The Lancet Neurology.

[175] Chiang HL, Lin CH (2019). Altered gut microbiome and intestinal pathology in Parkinson's disease. Journal of movement disorders.

[176] Ulusoy A, Phillips RJ, Helwig M, Klinkenberg M, Powley TL, Di Monte DA (2017). Brain-to-stomach transfer of alpha-synuclein via vagal preganglionic projections. Acta neuropathologica.

[177] Villaran RF, Espinosa-Oliva AM, Sarmiento M, De Pablos RM, Arguelles S, Delgado-Cortes MJ, Sobrino V, Van Rooijen N, Venero JL, Herrera AJ (2010). Ulcerative colitis exacerbates lipopolysaccharide-induced damage to the nigral dopaminergic system: potential risk factor in Parkinson`s disease. Journal of neurochemistry.

[178] Forsyth CB, Shannon KM, Kordower JH, Voigt RM, Shaikh M, Jaglin JA, Estes JD, Dodiya HB, Keshavarzian A (2011). Increased intestinal permeability correlates with sigmoid mucosa alpha-synuclein staining and endotoxin exposure markers in early Parkinson's disease. PloS one.

[179] Salat-Foix D, Tran K, Ranawaya R, Meddings J, Suchowersky O (2012). Increased intestinal permeability and Parkinson disease patients: chicken or egg?. The Canadian journal of neurological sciences Le journal canadien des sciences neurologiques.

[180] Keshavarzian A, Green SJ, Engen PA, Voigt RM, Naqib A, Forsyth CB, Mutlu E, Shannon KM (2015). Colonic bacterial composition in Parkinson's disease. Movement disorders : official journal of the Movement Disorder Society.

[181] Scheperjans F, Aho V, Pereira PA, Koskinen K, Paulin L, Pekkonen E, Haapaniemi E, Kaakkola S, Eerola-Rautio J, Pohja M (2015). Gut microbiota are related to Parkinson's disease and clinical phenotype. Movement disorders : official journal of the Movement Disorder Society.

[182] Barichella M, Pacchetti C, Bolliri C, Cassani E, Iorio L, Pusani C, Pinelli G, Privitera G, Cesari I, Faierman SA (2016). Probiotics and prebiotic fiber for constipation associated with Parkinson disease: an RCT. Neurology.

[183] Peter I, Dubinsky M, Bressman S, Park A, Lu C, Chen N, Wang A (2018). Anti-tumor necrosis factor therapy and incidence of Parkinson disease among patients with inflammatory bowel disease. JAMA neurology.

[184] Camacho-Soto A, Gross A, Searles Nielsen S, Dey N, Racette BA (2018). Inflammatory bowel disease and risk of Parkinson's disease in Medicare beneficiaries. Parkinsonism & related disorders.

[185] Alzheimer's A (2016). 2016 Alzheimer's disease facts and figures. Alzheimer's & dementia : the journal of the Alzheimer's Association.

[186] Wang MM, Miao D, Cao XP, Tan L, Tan L (2018). Innate immune activation in Alzheimer's disease. Annals of translational medicine.

[187] Bagyinszky E, Giau VV, Shim K, Suk K, An SSA, Kim S (2017). Role of inflammatory molecules in the Alzheimer's disease progression and diagnosis. Journal of the neurological sciences.

[188] Asti A, Gioglio L (2014). Can a bacterial endotoxin be a key factor in the kinetics of amyloid fibril formation?. Journal of Alzheimer's disease : JAD.

[189] Allen HB (2016). Alzheimer's disease: assessing the role of Spirochetes, biofilms, the immune system, and amyloid-beta with regard to potential treatment and prevention. Journal of Alzheimer's disease : JAD.

[190] Schwartz K, Boles BR (2013). Microbial amyloids--functions and interactions within the host. Current opinion in microbiology.

[191] Zhou Y, Smith D, Leong BJ, Brannstrom K, Almqvist F, Chapman MR (2012). Promiscuous cross-seeding between bacterial amyloids promotes interspecies biofilms. The Journal of biological chemistry.

[192] Hill JM, Lukiw WJ (2015). Microbial-generated amyloids and Alzheimer's disease (AD). Frontiers in aging neuroscience.

[193] Roubaud-Baudron C, Krolak-Salmon P, Quadrio I, Megraud F, Salles N (2012). Impact of chronic Helicobacter pylori infection on Alzheimer's disease: preliminary results. Neurobiology of aging.

[194] Bu XL, Yao XQ, Jiao SS, Zeng F, Liu YH, Xiang Y, Liang CR, Wang QH, Wang X, Cao HY (2015). A study on the association between infectious burden and Alzheimer's disease. European journal of neurology.

[195] Wang XL, Zeng J, Yang Y, Xiong Y, Zhang ZH, Qiu M, Yan X, Sun XY, Tuo QZ, Liu R (2015). Helicobacter pylori filtrate induces Alzheimer-like tau hyperphosphorylation by activating glycogen synthase kinase-3beta. JAD.

[196] Friedland RP, Chapman MR (2017). The role of microbial amyloid in neurodegeneration. PLoS pathogens.

[197] Schwartz K, Syed AK, Stephenson RE, Rickard AH, Boles BR (2012). Functional amyloids composed of phenol soluble modulins stabilize Staphylococcus aureus biofilms. PLoS pathogens.

[198] Harach T, Marungruang N, Duthilleul N, Cheatham V, Mc Coy KD, Frisoni G, Neher JJ, Fak F, Jucker M, Lasser T (2017). Reduction of Abeta amyloid pathology in APPPS1 transgenic mice in the absence of gut microbiota. Scientific reports.

[199] Al-Atrache Z, Lopez DB, Hingley ST, Appelt DM (2019). Astrocytes infected with Chlamydia pneumoniae demonstrate altered expression and activity of secretases involved in the generation of beta-amyloid found in Alzheimer disease. BMC neuroscience.

[200] Athari Nik Azm S, Djazayeri A, Safa M, Azami K, Ahmadvand B, Sabbaghziarani F, Sharifzadeh M, Vafa M (2018). Lactobacilli and bifidobacteria ameliorate memory and learning deficits and oxidative stress in beta-amyloid (1-42) injected rats. Applied physiology, nutrition, and metabolism = Physiologie appliquee, nutrition et metabolisme.

[201] Wang T, Hu X, Liang S, Li W, Wu X, Wang L, Jin F (2015). Lactobacillus fermentum NS9 restores the antibiotic induced physiological and psychological abnormalities in rats. Beneficial microbes.

[202] Liang S, Wang T, Hu X, Luo J, Li W, Wu X, Duan Y, Jin F (2015). Administration of Lactobacillus helveticus NS8 improves behavioral, cognitive, and biochemical aberrations caused by chronic restraint stress. Neuroscience.

[203] Akbari E, Asemi Z, Daneshvar Kakhaki R, Bahmani F, Kouchaki E, Tamtaji OR, Hamidi GA, Salami M (2016). Effect of probiotic supplementation on cognitive function and metabolic status in Alzheimer's disease: a randomized, double-blind and controlled trial. Frontiers in aging neuroscience.

[204] Kountouras J, Boziki M, Gavalas E, Zavos C, Grigoriadis N, Deretzi G, Tzilves D, Katsinelos P, Tsolaki M, Chatzopoulos D (2009). Eradication of Helicobacter pylori may be beneficial in the management of Alzheimer's disease. Journal of neurology.

[205] Abraham D, Feher J, Scuderi GL, Szabo D, Dobolyi A, Cservenak M, Juhasz J, Ligeti B, Pongor S, Gomez-Cabrera MC (2019). Exercise and probiotics attenuate the development of Alzheimer's disease in transgenic mice: role of microbiome. Experimental gerontology.

[206] Alonso A, Logroscino G, Jick SS, Hernan MA (2009). Incidence and lifetime risk of motor neuron disease in the United Kingdom: a population-based study. European journal of neurology.

[207] Wang MD, Little J, Gomes J, Cashman NR, Krewski D (2017). Identification of risk factors associated with onset and progression of amyotrophic lateral sclerosis using systematic review and meta-analysis. Neurotoxicology.

[208] Nguyen MD, D'Aigle T, Gowing G, Julien JP, Rivest S (2004). Exacerbation of motor neuron disease by chronic stimulation of innate immunity in a mouse model of amyotrophic lateral sclerosis. The Journal of neuroscience : the official journal of the Society for Neuroscience.

[209] Zhang R, Miller RG, Gascon R, Champion S, Katz J, Lancero M, Narvaez A, Honrada R, Ruvalcaba D, McGrath MS (2009). Circulating endotoxin and systemic immune activation in sporadic amyotrophic lateral sclerosis (sALS). Journal of neuroimmunology.

[210] Longstreth WT, Meschke JS, Davidson SK, Smoot LM, Smoot JC, Koepsell TD (2005). Hypothesis: a motor neuron toxin produced by a clostridial species residing in gut causes ALS. Medical hypotheses.

[211] Kaneko K, Hachiya NS (2006). Hypothesis: gut as source of motor neuron toxin in the development of ALS. Medical hypotheses.

[212] Al-Asmakh M, Hedin L (2015). Microbiota and the control of blood-tissue barriers. Tissue barriers.

[213] Wu Shaoping, Yi Jianxun, Zhang Yong-guo, Zhou Jingsong, Sun Jun (2015). Leaky intestine and impaired microbiome in an amyotrophic lateral sclerosis mouse model. Physiological Reports.

[214] Fang X, Wang X, Yang S, Meng F, Wang X, Wei H, Chen T (2016). Evaluation of the microbial diversity in amyotrophic lateral sclerosis using high-throughput sequencing. Frontiers in microbiology.

[215] Brenner D, Hiergeist A, Adis C, Mayer B, Gessner A, Ludolph AC, Weishaupt JH (2018). The fecal microbiome of ALS patients. Neurobiology of aging.

[216] Zhang YG, Wu S, Yi J, Xia Y, Jin D, Zhou J, Sun J (2017). Target intestinal microbiota to alleviate disease progression in amyotrophic lateral sclerosis. Clinical therapeutics.

[217] Mazzini L, Mogna L, De Marchi F, Amoruso A, Pane M, Aloisio I, Cionci NB, Gaggia F, Lucenti A, Bersano E, et al. Potential role of gut microbiota in ALS pathogenesis and possible novel therapeutic strategies, Journal of clinical gastroenterology. 52 Suppl 1, Proceedings from the 9th Probiotics, Prebiotics and New Foods, Nutraceuticals and Botanicals for Nutrition & Human and Microbiota Health Meeting, held in Rome, Italy from September 10 to 12, 2017:2018, S68–S70. 10.1097/MCG.0000000000001042 29782468

[218] Tremlett H, Bauer KC, Appel-Cresswell S, Finlay BB, Waubant E (2017). The gut microbiome in human neurological disease: a review. Annals of neurology.

[219] Chen J, Chia N, Kalari KR, Yao JZ, Novotna M, Paz Soldan MM, Luckey DH, Marietta EV, Jeraldo PR, Chen X (2016). Multiple sclerosis patients have a distinct gut microbiota compared to healthy controls. Scientific reports.

[220] Buscarinu MC, Cerasoli B, Annibali V, Policano C, Lionetto L, Capi M, Mechelli R, Romano S, Fornasiero A, Mattei G (2017). Altered intestinal permeability in patients with relapsing-remitting multiple sclerosis: A pilot study. Multiple sclerosis.

[221] Dendrou CA, Fugger L, Friese MA (2015). Immunopathology of multiple sclerosis. Nat Rev Immunol.

[222] Chu F, Shi M, Lang Y, Shen D, Jin T, Zhu J, Cui L (2018). Gut microbiota in multiple sclerosis and experimental autoimmune encephalomyelitis: current applications and future perspectives. Mediators of inflammation.

[223] Ezendam J, de Klerk A, Gremmer ER, van Loveren H (2008). Effects of Bifidobacterium animalis administered during lactation on allergic and autoimmune responses in rodents. Clinical and experimental immunology.

[224] Takata K, Kinoshita M, Okuno T, Moriya M, Kohda T, Honorat JA, Sugimoto T, Kumanogoh A, Kayama H, Takeda K (2011). The lactic acid bacterium Pediococcus acidilactici suppresses autoimmune encephalomyelitis by inducing IL-10-producing regulatory T cells. PloS one.

[225] Tremlett H, Fadrosh DW, Faruqi AA, Hart J, Roalstad S, Graves J, Lynch S, Waubant E (2016). Centers USNoPM: Gut microbiota composition and relapse risk in pediatric MS: a pilot study. Journal of the neurological sciences.

[226] Adamczyk-Sowa M, Medrek A, Madej P, Michlicka W, Dobrakowski P (2017). Does the gut microbiota influence immunity and inflammation in multiple sclerosis pathophysiology?. Journal of immunology research.

[227] Ochoa-Reparaz J, Mielcarz DW, Ditrio LE, Burroughs AR, Foureau DM, Haque-Begum S, Kasper LH (2009). Role of gut commensal microflora in the development of experimental autoimmune encephalomyelitis. Journal of immunology.

[228] Bron PA, Kleerebezem M, Brummer RJ, Cani PD, Mercenier A, MacDonald TT, Garcia-Rodenas CL, Wells JM (2017). Can probiotics modulate human disease by impacting intestinal barrier function?. The British journal of nutrition.

[229] Wing AC, Kremenchutzky M (2019). Multiple sclerosis and faecal microbiome transplantation: are you going to eat that?. Beneficial microbes.

[230] Fernandes CP, Oliveira FA, Silva PG, Alves AP, Mota MR, Montenegro RC, Burbano RM, Seabra AD, Lobo Filho JG, Lima DL (2014). Molecular analysis of oral bacteria in dental biofilm and atherosclerotic plaques of patients with vascular disease. International journal of cardiology.

[231] Apfalter P, Hammerschlag MR, Boman J (2003). Reliability of nested PCR for the detection of Chlamydia pneumoniae in carotid artery atherosclerosis. Stroke.

[232] Mitra S, Drautz-Moses DI, Alhede M, Maw MT, Liu Y, Purbojati RW, Yap ZH, Kushwaha KK, Gheorghe AG, Bjarnsholt T (2015). In silico analyses of metagenomes from human atherosclerotic plaque samples. Microbiome.

[233] Karlsson FH, Fak F, Nookaew I, Tremaroli V, Fagerberg B, Petranovic D, Backhed F, Nielsen J (2012). Symptomatic atherosclerosis is associated with an altered gut metagenome. Nature communications.

[234] Ufnal M, Zadlo A, Ostaszewski R (2015). TMAO: a small molecule of great expectations. Nutrition.

[235] Skagen K, Troseid M, Ueland T, Holm S, Abbas A, Gregersen I, Kummen M, Bjerkeli V, Reier-Nilsen F, Russell D (2016). The carnitine-butyrobetaine-trimethylamine-N-oxide pathway and its association with cardiovascular mortality in patients with carotid atherosclerosis. Atherosclerosis.

[236] Lau K, Srivatsav V, Rizwan A, Nashed A, Liu R, Shen R, Akhtar M. Bridging the gap between gut microbial dysbiosis and cardiovascular diseases. Nutrients. 2017:9(8). 10.3390/nu9080859 PMC5579652 28796176

[237] Tang WHW, Backhed F, Landmesser U, Hazen SL (2019). Intestinal microbiota in cardiovascular health and disease: JACC State-of-the-Art Review. Journal of the American College of Cardiology.

[238] Kiessling G, Schneider J, Jahreis G (2002). Long-term consumption of fermented dairy products over 6 months increases HDL cholesterol. European journal of clinical nutrition.

[239] Rerksuppaphol S, Rerksuppaphol L (2015). A randomized double-blind controlled trial of Lactobacillus acidophilus plus Bifidobacterium bifidum versus placebo in patients with hypercholesterolemia. Journal of clinical and diagnostic research : JCDR.

[240] Qiu L, Tao X, Xiong H, Yu J, Wei H (2018). Lactobacillus plantarum ZDY04 exhibits a strain-specific property of lowering TMAO via the modulation of gut microbiota in mice. Food & function.

[241] Benakis C, Brea D, Caballero S, Faraco G, Moore J, Murphy M, Sita G, Racchumi G, Ling L, Pamer EG (2016). Commensal microbiota affects ischemic stroke outcome by regulating intestinal gammadelta T cells. Nature medicine.

[242] Pussinen PJ, Alfthan G, Jousilahti P, Paju S, Tuomilehto J (2007). Systemic exposure to Porphyromonas gingivalis predicts incident stroke. Atherosclerosis.

[243] Kawato T, Tanaka H, Tabuchi M, Ooshima K, Nakai K, Yamashita Y, Maeno M (2013). Continual Gram-negative bacterial challenge accelerates stroke onset in stroke-prone spontaneously hypertensive rats. Clinical and experimental hypertension.

[244] Zhu W, Wang Z, Tang WHW, Hazen SL (2017). Gut microbe-generated trimethylamine N-oxide from dietary choline is prothrombotic in subjects. Circulation.

[245] Yin J, Liao SX, He Y, Wang S, Xia GH, Liu FT, Zhu JJ, You C, Chen Q, Zhou L, et al. Dysbiosis of gut microbiota with reduced trimethylamine-N-oxide level in patients with large-artery atherosclerotic stroke or transient ischemic attack. Journal of the American Heart Association. 2015:4(11). 10.1161/JAHA.115.002699 PMC4845212 26597155

[246] Shikata F, Shimada K, Sato H, Ikedo T, Kuwabara A, Furukawa H, Korai M, Kotoda M, Yokosuka K, Makino H (2019). Potential influences of gut microbiota on the formation of intracranial aneurysm. Hypertension.

[247] Fischer A, Zalvide J, Faurobert E, Albiges-Rizo C, Tournier-Lasserve E (2013). Cerebral cavernous malformations: from CCM genes to endothelial cell homeostasis. Trends in molecular medicine.

[248] Tang AT, Choi JP, Kotzin JJ, Yang Y, Hong CC, Hobson N, Girard R, Zeineddine HA, Lightle R, Moore T (2017). Endothelial TLR4 and the microbiome drive cerebral cavernous malformations. Nature.

[249] Winek K, Engel O, Koduah P, Heimesaat MM, Fischer A, Bereswill S, Dames C, Kershaw O, Gruber AD, Curato C (2016). Depletion of cultivatable gut microbiota by broad-spectrum antibiotic pretreatment worsens outcome after murine stroke. Stroke.

[250] Boumis E, Capone A, Galati V, Venditti C, Petrosillo N (2018). Probiotics and infective endocarditis in patients with hereditary hemorrhagic` telangiectasia: a clinical case and a review of the literature. BMC Infect Dis.

[251] Sun Yundong, Zhang Min, Chen Chun–Chia, Gillilland Merritt, Sun Xia, El–Zaatari Mohamad, Huffnagle Gary B., Young Vincent B., Zhang Jiajie, Hong Soon–Cheol, Chang Yu–Ming, Gumucio Deborah L., Owyang Chung, Kao John Y. (2013). Stress-Induced Corticotropin-Releasing Hormone-Mediated NLRP6 Inflammasome Inhibition and Transmissible Enteritis in Mice. Gastroenterology.

[252] Falony G, Joossens M, Vieira-Silva S, Wang J, Darzi Y, Faust K, Kurilshikov A, Bonder MJ, Valles-Colomer M, Vandeputte D (2016). Population-level analysis of gut microbiome variation. Science.

[253] Rothschild D, Weissbrod O, Barkan E, Kurilshikov A, Korem T, Zeevi D, Costea PI, Godneva A, Kalka IN, Bar N (2018). Environment dominates over host genetics in shaping human gut microbiota. Nature.

[254] Zhernakova A, Kurilshikov A, Bonder MJ, Tigchelaar EF, Schirmer M, Vatanen T, Mujagic Z, Vila AV, Falony G, Vieira-Silva S (2016). Population-based metagenomics analysis reveals markers for gut microbiome composition and diversity. Science.

[255] Jonsson AL, Backhed F (2017). Role of gut microbiota in atherosclerosis. Nature reviews Cardiology.

[256] Lin S, Lin Y, Nery JR, Urich MA, Breschi A, Davis CA, Dobin A, Zaleski C, Beer MA, Chapman WC (2014). Comparison of the transcriptional landscapes between human and mouse tissues. Proceedings of the National Academy of Sciences of the United States of America.

[257] Mouse Genome Sequencing C, Waterston RH, Lindblad-Toh K, Birney E, Rogers J, Abril JF, Agarwal P, Agarwala R, Ainscough R, Alexandersson M (2002). Initial sequencing and comparative analysis of the mouse genome. Nature.

[258] Church DM, Goodstadt L, Hillier LW, Zody MC, Goldstein S, She X, Bult CJ, Agarwala R, Cherry JL, DiCuccio M (2009). Lineage-specific biology revealed by a finished genome assembly of the mouse. PLoS biology.

[259] Hugenholtz F, de Vos WM (2018). Mouse models for human intestinal microbiota research: a critical evaluation. Cellular and molecular life sciences : CMLS.

[260] Scholtens PA, Oozeer R, Martin R, Amor KB, Knol J (2012). The early settlers: intestinal microbiology in early life. Annual review of food science and technology.

[261] Zeevi D, Korem T, Zmora N, Israeli D, Rothschild D, Weinberger A, Ben-Yacov O, Lador D, Avnit-Sagi T, Lotan-Pompan M (2015). Personalized Nutrition by Prediction of Glycemic Responses. Cell.

[262] Costea PI, Zeller G, Sunagawa S, Pelletier E, Alberti A, Levenez F, Tramontano M, Driessen M, Hercog R, Jung FE (2017). Towards standards for human fecal sample processing in metagenomic studies. Nature biotechnology.

[263] Franzosa EA, Morgan XC, Segata N, Waldron L, Reyes J, Earl AM, Giannoukos G, Boylan MR, Ciulla D, Gevers D (2014). Relating the metatranscriptome and metagenome of the human gut. Proceedings of the National Academy of Sciences of the United States of America.

[264] Sinha R, Abu-Ali G, Vogtmann E, Fodor AA, Ren B, Amir A, Schwager E, Crabtree J, Ma S (2017). Microbiome Quality Control Project C et al: Assessment of variation in microbial community amplicon sequencing by the Microbiome Quality Control (MBQC) project consortium. Nature biotechnology.

[265] Shah N, Tang H, Doak TG, Ye Y. Comparing bacterial communities inferred from 16S rRNA gene sequencing and shotgun metagenomics. Pacific Symposium on Biocomputing Pacific Symposium on Biocomputing. 2011:165–76. 10.1142/9789814335058_0018 21121044

[266] Steven B, Gallegos-Graves LV, Starkenburg SR, Chain PS, Kuske CR (2012). Targeted and shotgun metagenomic approaches provide different descriptions of dryland soil microbial communities in a manipulated field study. Environmental microbiology reports.

[267] Poretsky R, Rodriguez RL, Luo C, Tsementzi D, Konstantinidis KT (2014). Strengths and limitations of 16S rRNA gene amplicon sequencing in revealing temporal microbial community dynamics. PloS one.

[268] Meisel JS, Hannigan GD, Tyldsley AS, SanMiguel AJ, Hodkinson BP, Zheng Q, Grice EA (2016). Skin microbiome surveys are strongly influenced by experimental design. The Journal of investigative dermatology.

[269] Scholz M, Ward DV, Pasolli E, Tolio T, Zolfo M, Asnicar F, Truong DT, Tett A, Morrow AL, Segata N (2016). Strain-level microbial epidemiology and population genomics from shotgun metagenomics. Nature methods.

[270] Winek K, Meisel A, Dirnagl U (2016). Gut microbiota impact on stroke outcome: fad or fact?. Journal of cerebral blood flow and metabolism : official journal of the International Society of Cerebral Blood Flow and Metabolism.

